# Curtailment of Toll-like receptor signalling and cytokine production in dendritic cells by secreted products of *Heligmosomoides polygyrus*

**DOI:** 10.1371/journal.ppat.1014528

**Published:** 2026-08-26

**Authors:** Andrea M. Kemter, John R. Grainger, Danielle J. Smyth, Cecilia Forss, Alexander Phythian-Adams, Ruby F. White, Arianna Raponi, Alasdair C. Ivens, Andrew S. MacDonald, Henry J. McSorley, Rick M. Maizels

**Affiliations:** 1 Centre for Parasitology, School of Infection and Immunity, University of Glasgow, United Kingdom; 2 Institute of Immunology and Infection Research, School of Biological Sciences, University of Edinburgh, United Kingdom; 3 Medical Research Council Centre of Research Excellence in Exposome Immunology, United Kingdom; 4 Lydia Becker Institute of Immunology and Inflammation, Faculty of Biology, Medicine and Health, University of Manchester, United Kingdom; Rowan University, UNITED STATES OF AMERICA

## Abstract

Dendritic cells (DCs) are the professional antigen-presenting cells responsible for recognition of pathogens and induction of appropriate adaptive immune responses. Many successful pathogens have evolved to release molecular products that can manipulate DC responses to dampen immunity. Here, we report that the excretory/secretory (ES) products of the intestinal helminth *Heligmosomoides polygyrus bakeri* profoundly repress the ability of murine and human DCs, differentiated under various conditions, to respond to canonical stimuli delivered through Toll-like Receptors (TLRs). *H. polygyrus* ES (HES) blocks production of pro-inflammatory cytokines, especially IL-6 and IL-12p70, at both the mRNA and secreted protein levels. In addition, upregulation of the costimulatory molecules CD40, CD80 and CD86 in response to LPS and other TLR ligands is inhibited. Although HES contains a mimic of host TGF-β, inhibition of DC activation is mediated independently of TGF-β signalling and by a HES protein fraction devoid of TGF-β-like proteins; we were not able to recapitulate inhibition with recombinant forms of 5 proteins identified in this fraction. HES pre-treatment of DCs blocks subsequent LPS-induced activation, excluding any direct interference with ligand binding. Inhibition can also be observed if HES is added up to 8 hours after LPS treatment, indicating modulation of later phase intracellular pathways. Analysis of activation of the NF-κB and MAPK signalling pathways and the time courses of CD40, IL-6, IL-12 and TNF expression confirms that HES does not affect the immediate-early phase of TLR-induced activation, but interferes with the ability of DCs to sustain activation and complete the inflammatory response programme, with IL-12 the most susceptible to inhibition as its production is initiated later than that of IL-6 and TNF. These data indicate finely staged changes in the maturation process of DCs upon HES treatment that have not previously been reported, helping explain how parasites like *H. polygyrus* are able to dramatically modulate host immunity.

## Author summary

Helminth parasites are masters of immune evasion. To forestall an effective immune response, we hypothesise that they manipulate the pivotal inducer cells of the immune system, dendritic cells (DCs) that recognise pathogen patterns and activate host lymphocytes to attack the parasite. We studied the intestinal helminth *Heligmosomoides polygyrus* which is a natural parasite of mice; proteins secreted by this worm were found to interfere with normal DC responses, such as the production of the inflammatory cytokines IL-6 and IL-12, in response to bacterial products such as lipopolysaccharide (LPS). DCs also upregulate key surface proteins such as CD40, CD80 and CD86 in response to LPS, and the helminth products also inhibit these. We studied the kinetics of inhibition and show that while the early phase of the response (up to 8 hrs) is not down-modulated, the effects of helminth secretions then cut short the ability of DCs to produce inflammatory cytokines, in particular IL-12 which is the key spark of inflammation. By preventing sustained activation of DCs, the normal inflammatory programme is interrupted, and the ability of DCs to drive immunity is compromised.

## Introduction

To evade the immune system of the host, helminths have evolved a spectrum of sophisticated mechanisms to inhibit immune reactions by blocking immune recognition, activation and effector pathways [1–3]. Dendritic cells (DCs) are the pivotal professional antigen-presenting cells that both activate and shape adaptive immune responses [4–6] and are therefore prime targets for helminth immunomodulation. While the role of DCs in driving immune responses to helminths is well-documented [7–13], with emphasis on the cDC2 subset that induces type 2 immunity [5,6,14,15] our understanding of how helminths may interfere with DC functions remains incomplete. In part, this is due to few defined pathogen associated molecular patterns (PAMPs) from these parasites, and reflects the need for more mechanistic investigations of helminth modulatory effects on DCs [16–19]. Helminth interference with DC function not only impacts on the development of immune reactions to the helminth itself, but also on responses to bystander antigens or co-infections [11,19,20].

Normally, when DCs encounter PAMPs such as lipopolysaccharide (LPS), a number of signalling pathways are activated to induce the maturation of these cells. The signalling pathways downstream of TLRs converge with the activation of TRAF6, which in turn induces the activation of, amongst others, NF-κB and the MAPK cascade. For NF-κB induction, activation of IKKs leads to phosphorylation of IκBs, targeting them for proteasomal degradation. This frees previously IκB-bound NF-κB to translocate to the nucleus and induce transcription of target genes [21]. The MAPK cascade involves sequential phosphorylation of target proteins by several kinases, with the three branches named after the final kinases in the cascades, p38, ERK and JNK. These phosphorylate components of the AP-1 transcription factor, thereby inducing its activation [22,23]. Signalling through these pathways finally leads to production and secretion of cytokines as well as upregulation of costimulatory molecules, the hallmarks of DC activation.

Helminths, however, not only induce altered DC activation states, but can also actively interfere with TLR ligand-induced DC activation in a variety of ways [1,24]. Components of the trematode *Fasciola hepatica* for example can inhibit activation of the NF-κB subunit p65 [25] as well as MAPKs [26]. Omega-1, a ribonuclease responsible for some of the immune modulatory effects of the soluble egg antigens from *Schistosoma mansoni* (SEA) is another prominent example. This protein induces a T_H_2 response upon injection into naive mice and inhibits LPS-induced upregulation of CD86 and production of IL-12p70 by human monocyte derived DCs (moDC). Omega-1 function is dependent on both its glycosylation and its activity as an RNase, as it is bound and internalized by the mannose receptor on moDC and subsequently non-specifically degrades both rRNA and mRNA, effectively inhibiting protein synthesis by DCs [27–29]. Another well characterized glycoprotein inhibiting DC function is ES-62, a phosphorylcholine-bearing excretory/secretory (ES) product of the filarial nematode *Acanthocheilonema viteae* [30], which sequesters the MyD88 signalling protein [31] and induces autophagic degradation of key components of LPS-induced pathways [32]. The ES of *Trichuris suis,* a nematode that has been used in a number of clinical trials of live helminth therapy [33], can inhibit signalling pathways downstream of TLR4, as well as actively reduce the expression levels of TLR4 itself [34,35].

Two widely used model organisms for hookworm infections, *Nippostrongylus brasiliensis* as well as *Heligmosomoides polygyrus bakeri,* also secrete products that modulate DC activation [36,37]. Mouse bone marrow-derived DCs (BMDCs) treated with *H. polygyrus* ES (HES), but not those treated with worm extracts, showed reduced expression of costimulatory molecules and proinflammatory cytokines after stimulation with CpG and LPS, while HES-treatment of ovalbumin peptide-pulsed DCs reduced their ability to induce antibody responses when transferred *in vivo* [37].

Infection with *H. polygyrus* also modulates intestinal DCs, expanding a population of CD11c^lo^ DCs in mesenteric lymph nodes (MLNs) that preferentially induced Tregs rather than effector T cells [8,38], while Th2 responses were favoured by MLN cDC2s defined by CD11b expression [39]. MLN DCs from infected mice also reacted more poorly to TLR ligands such as LPS and polyinosinic-polycytidylic acid (poly-IC) [8]. Within the lamina propria of infected mice, DCs express lower levels of costimulatory molecules CD80 and CD86 [40] and the C-type lectin dectin-1 [41], and were less able to induce production of IFNγ and IL-17 upon co-culture with T cells [41]. Adoptive transfer of MLN DCs from *H. polygyrus*-infected mice reduced colitic inflammation in a T-cell transfer model of colitis with reduced cytokine responses from co-transferred ovalbumin (OVA)-specific OT-II T cells [42]. Likewise, *H. polygyrus*-experienced DCs, when transferred into mice at the time of *Citrobacter rodentium*-infect infection, compromised anti-bacterial immunity in a manner dependent on DC IL-10 expression [43]. Recent phenotyping of lamina propria cDC2s from *H. polygyrus*-infected mice distinguished two functional subsets with CD103^+^ cDC2s favouring Treg differentiation, while CD103^–^ cDC2s induced stronger T cell cytokine responses [44], reflecting the competing pathways activated during a live helminth infection.

Here, we focus on the effects of parasite products, rather than active infection, on DC phenotype and function. We found that HES broadly inhibits TLR ligand-induced DC activation, whether DCs were pre-treated with HES, HES was added at the time of stimulation or even if HES was added hours after stimulation. Furthermore, HES did not affect the immediate-early activation of signalling pathways or the initiation of DC activation; rather, it impacted the maintenance of MAPK and NF-kB signalling. This coincided with a loss of further cytokine production and a reduction of costimulatory molecule upregulation, leading to the observed inhibition of DC activation. These results provide a new insight into the effect of HES on DCs and advance us towards elucidating the mechanism by which HES affects TLR ligand-induced DC maturation.

## Results

### HES profoundly inhibits activation of dendritic cells by TLR ligands

**Fig 1:**
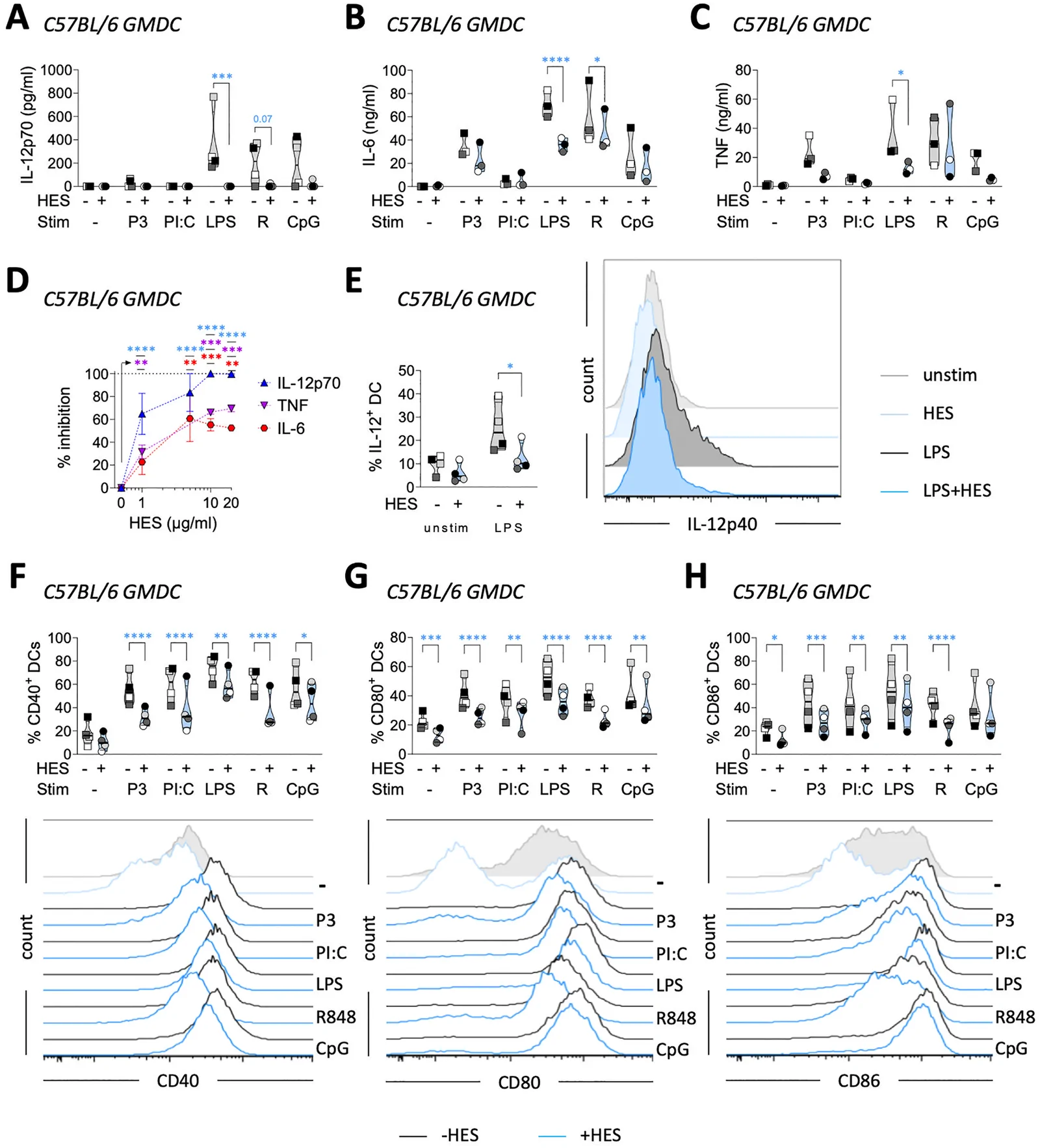
HES profoundly inhibits activation of dendritic cells by TLR ligands. GMDC of C57BL/6 mice were differentiated with GM-CSF for 10 days before incubation with TLR ligands Pam3CSK4 (P3, ligand for TLR1/2), Poly(I:C) (PI:C, TLR3), LPS (TLR4), R848 (R, TLR7/8) and CpG (TLR9) and HES for 18 h. A-C. Secretion of IL-12p70, IL-6 and TNF by GMDC stimulated as indicated in presence or absence of 10 µg/mL HES and measured by ELISA. D. Dose titration of HES in GMDC from C57BL/6 mice, measuring LPS-induced secretion of IL-12p70, IL-6 and TNF measured by ELISA. LPS-stimulated cells without HES served as 0% inhibition control, and unstimulated cells as 100%. E. Percentage of GMDC from C57BL/6 mice positive for intracellular IL-12p40 as measured by flow cytometry. F-H. Expression of CD40, CD80 and CD86 on CD11c^+^ cells determined by flow cytometry. Each symbol represents the mean of 3 samples from one experiment, symbol colour marking each independent experiment. n = 2–5 experiments. Results from 2-way repeated measures (RM) ANOVA matching both factors and Šídák’s multiple comparison test (or mixed effects model with Dunnet’s for D) are indicated as *: p ≤ 0.05; **: p ≤ 0.01; ***: p ≤ 0.001; ****: p ≤ 0.0001. Sources of variation for the 2-way ANOVA are compiled in Supplementary Information (Statistical and Data Annex).

The intestinal helminth *H. polygyrus* is able to reduce the severity of a range of inflammatory disorders in mouse models [45–49], and its effects can be largely replicated by administration of excretory-secretory (HES) products [50–52]. Although HES has been reported to inhibit activation of murine BMDCs by various TLR ligands [37], no systematic appraisal has yet been performed. We therefore first tested BMDCs from C57BL/6 mice differentiated with GM-CSF (GMDCs) before stimulation with the ligands Pam3CSK4 (TLR1/2), Poly(I:C) (TLR3), LPS (TLR4), R848 (TLR7/8) and CpG (TLR9) for 18 h, with or without the concurrent addition of HES. As can be seen in Fig 1A, secretion of the type 1-inducing cytokine IL-12p70 was induced by LPS, R848 and CpG, but in each case cytokine production was completely abrogated in the presence of 10 µg/mL HES. Several TLR ligands also induced significant release of IL-6; HES tended to inhibit the secretion of this cytokine, although only after stimulation with LPS and R848 did the reduction reach statistical significance (Fig 1B). Similarly, HES strongly abated the TNF response to LPS, with minor effects following stimulation with other ligands (Fig 1C), indicating a selective effect rather than any impact of HES on cell viability. All 3 cytokine responses to LPS showed parallel degrees of reduction over a dose-response titration of HES (Fig 1D), suggesting a common pathway of inhibition. In further experiments, GMDCs from BALB/c mice were found to be similarly susceptible to HES inhibition of inflammatory cytokine responses to a range of TLR ligands and doses of HES (S1 A, S1B Fig).

To investigate at which level HES inhibits the activation of DCs, C57BL/6 GMDCs were stimulated with LPS in the presence or absence of HES, and their production of IL-12 determined by intracellular cytokine staining followed by flow cytometry. Upon stimulation with LPS, the percentage of IL-12^+^ GMDCs increased as expected, but HES exerted a marked reduction in cytokine staining, indicating inhibition of cellular cytokine production and not only secretion into the environment (Fig 1E). Parallel results were found with HES inhibition of intracellular IL-12 expression in BALB/c GMDCs (S1 C Fig).

A corollary of DC activation is not only inflammatory cytokine production, but upregulation of key co-stimulatory surface markers such as CD40, CD80 and CD86, which are necessary to induce adaptive immune responses in B and T cells. Each of the 5 TLR ligands tested raised the intensity and frequency of CD40 (Fig 1F), CD80 (Fig 1G) and CD86 (Fig 1H) co-stimulator expression on C57BL/6 GMDCs as measured by flow cytometry; additional treatment with HES inhibited upregulation of all 3 markers with all stimuli tested. Again, we found similar suppressive effects of HES on co-stimulatory marker upregulation in BALB/c GMDCs (S1D Fig).

Within GMDCs, two different phenotypes have been discerned [53,54], one of which corresponds to conventional DCs, and a second macrophage-like type expressing the CD115 marker that is also associated with a cDC2B subset [55]. Following depletion of CD115^+^ cells by magnetic activated cell sorting (MACS) we compared the responses of total GMDCs and CD115-depleted cells to LPS and HES and found that, in both cases, IL-12p70 responses were completely abolished by HES, indicating that CD115^+^ cells alone could not account for the effects observed (S1E Fig).

### HES inhibits activation of both conventional and plasmacytoid DC subsets

**Fig 2:**
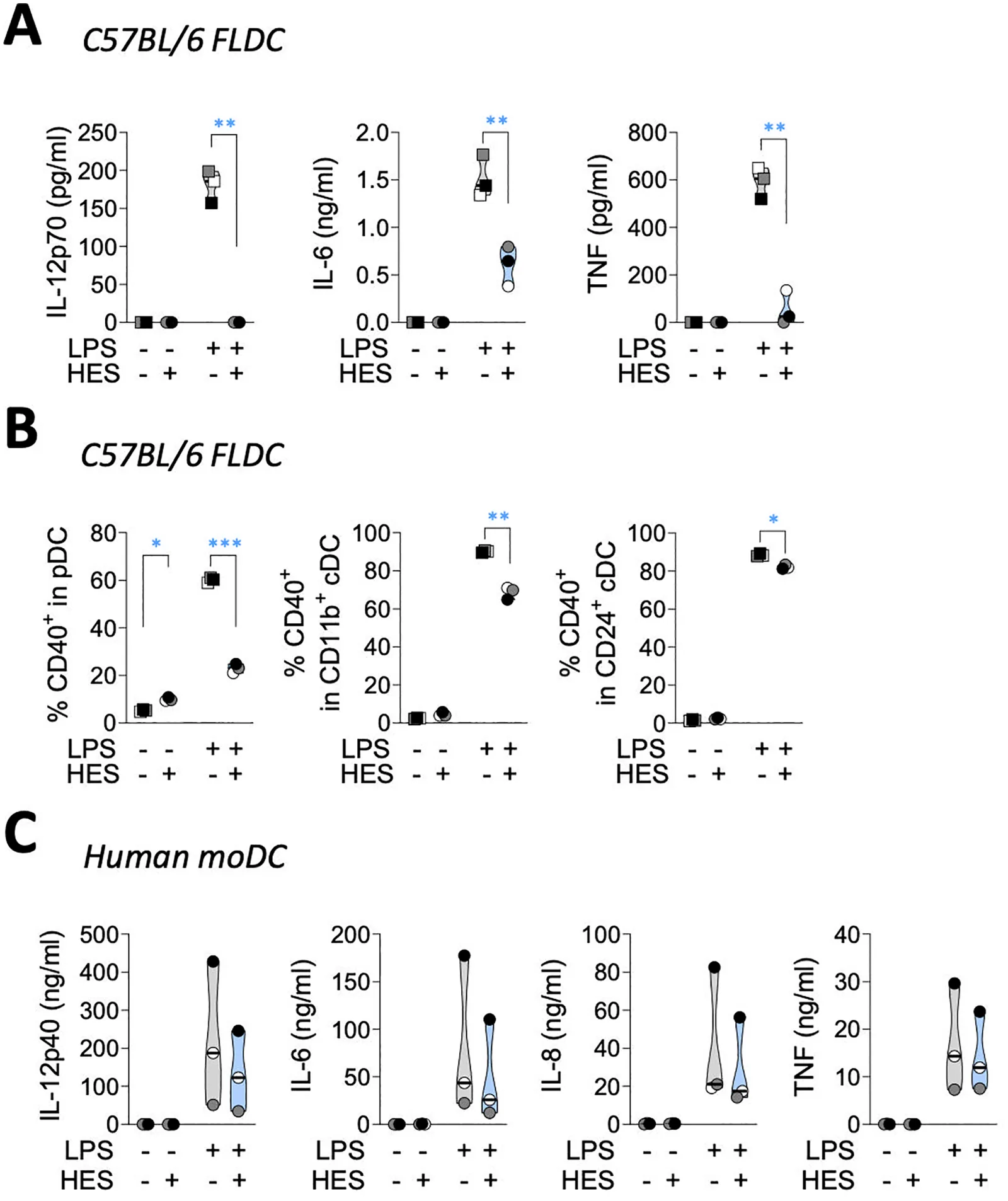
HES inhibits activation of multiple DC subsets in response to TLR ligation. A. FLDC were differentiated with Flt3-L for 8 days before incubation with LPS and HES for 18h as indicated. Concentrations of IL-12p70, IL-6 and TNF in the supernatants of total FLDC were measured by ELISA. Each symbol represents the mean of 2 samples from one bone marrow donor, symbol colour marking each separate donor, n = 3, shown is one of two separate experiments. B. FLDC from stimulation in A were analysed by flow cytometry to distinguish between different subsets and determine their expression of activation markers. pDC (left panel) were defined as B220^+^ cells, while B220^–^ cells were labelled cDC, sub-divided into CD11b^+^ (centre) and CD24^+^ (right panel) populations as defined elsewhere [57]. C. Human PBMC were differentiated to moDC with GM-CSF and IL-4 for six days before incubation with LPS and HES for 18 h as indicated. Concentrations of IL-12p40, IL-6, IL-8 and TNF in culture supernatants were analysed by CBA. Each symbol represents the mean of 2 samples from one donor, symbol colour marking each donor. n = 3 donors. Results from 2-way RM ANOVA matching both factors and Šídák’s multiple comparison test are indicated as *: p ≤ 0.05; **: p ≤ 0.01; ***: p ≤ 0.001; ****: p ≤ 0.0001. Sources of variation for the 2-way ANOVA are compiled in Supplementary Information (Statistical and Data Annex).

As an alternative to *in vitro* generation of GMDCs with GM-CSF, differentiation of murine bone marrow cultures with Flt3 ligand (Flt3-L) has been found to produce DCs (FLDCs) that more closely represent DC subsets found *in vivo*, including B220^+^ cells that are considered pDCs, and B220^−^ cells that resemble cDCs [56]. The latter cells can be further subdivided into CD24^+^ cells and CD11b^+^ cells, the former corresponding to cDC1s and the latter to cDC2s *in vivo* [57]. To investigate the effect HES has on these DC subsets, bone marrow cells were differentiated with Flt3-L before stimulation with LPS in the presence or absence of HES for 18h. Just as described for GMDCs (Fig 1), LPS stimulation induced secretion of IL-12p70, IL-6 and TNF by FLDCs. Again, IL-12p70 production was completely inhibited by HES. IL-6 and TNF concentrations in supernatants were also dramatically reduced (Fig 2A). In addition, the costimulatory molecule CD40 was highly induced in all subsets. As shown in Fig 2B, HES inhibited the upregulation of CD40 to varying degrees but significantly in all three subsets, with greatest impact on pDCs and only a slight inhibition of CD24^+^ cDC1s by HES.

As a further approach to ascertain the inhibitory effect of HES on DCs, murine splenocytes were isolated and enriched for CD11c^+^ cells by MACS, then stimulated for 18h with LPS ± HES. The LPS-induced secretion of IL-6 and TNF was reduced by HES co-culture (S2A Fig). IL-12p70 could not be detected in the culture supernatants.

A broader question of key interest was whether HES is also able to suppress activation of human DCs, which would indicate that the parasite is targeting a pathway conserved across mammalian host species. To examine this question, human peripheral blood monocytes were differentiated into monocyte derived DCs (moDCs) with GM-CSF and IL-4 for seven days before stimulation with LPS ± HES for 18h. Concentrations of IL-12p40, IL6, IL-8 and TNF in the culture supernatants were determined by cytokine bead array. Although moDCs from different donors displayed a high variability in the strength of their cytokine response, nevertheless, in each donor HES reduced the levels of IL-12p40 and IL-6 secreted following LPS stimulation moDCs (Fig 2C). The expression of costimulatory molecules on CD14^−^CD11c^+^ cells, however, was not affected by HES, as is shown in S2B Fig for CD80.

### HES induces a subtle but distinctive change in GMDC gene expression

**Fig 3:**
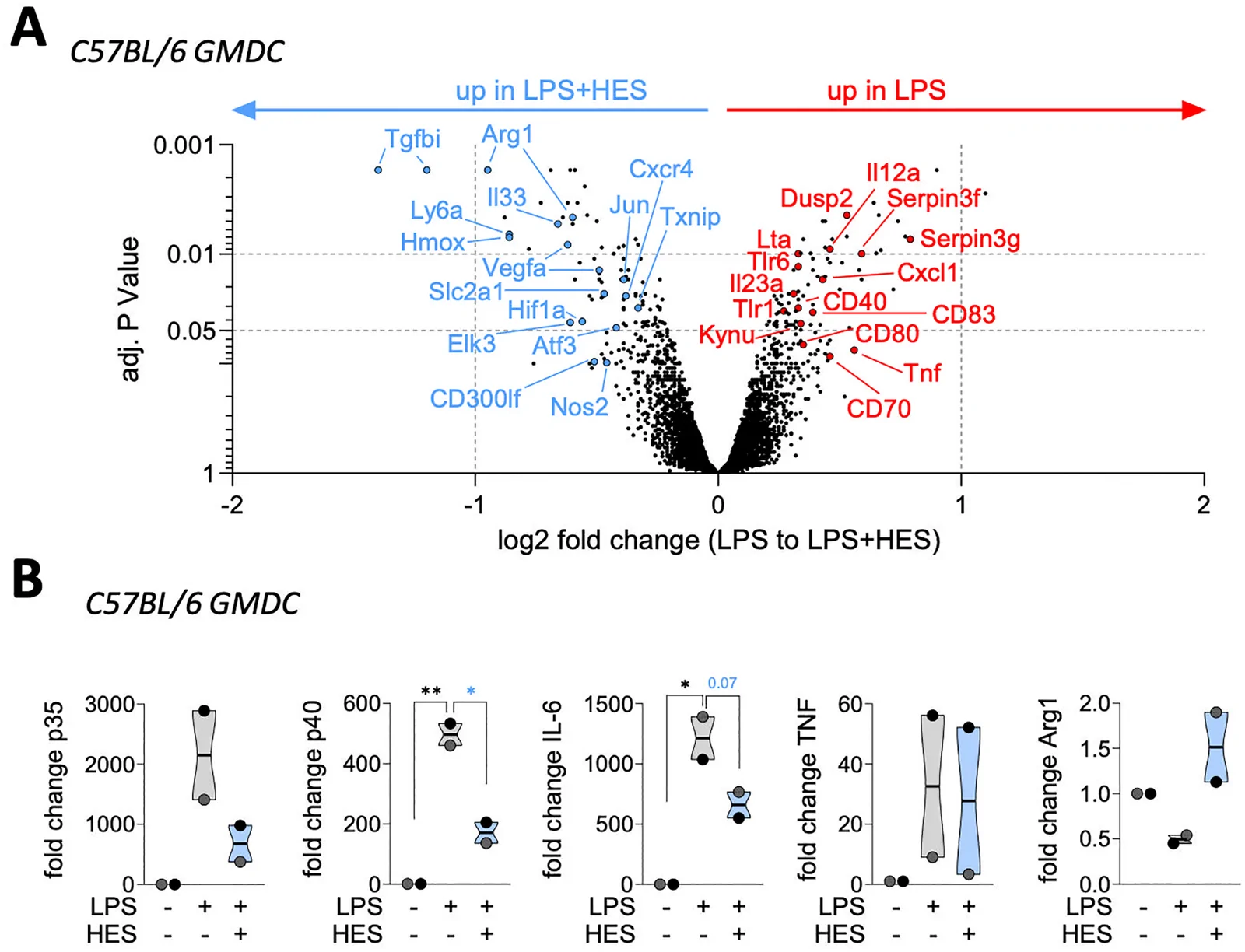
HES induces a subtle but distinctive change in GMDC gene expression. A. Gene expression changes in C57BL/6 GMDC after 8 h of LPS-stimulation with or without HES were measured by microarray. Shown are average values of 3 samples from one experiment. Significance of fold changes was controlled for false discovery, shown are adjusted p values. B. Levels of IL-12p35 and p40, IL-6, TNF and Arginase-1 mRNA were measured by quantitative real-time PCR at 8 h after stimulation and normalized to Rpl13a as a housekeeping gene and the unstimulated control groups using the ΔΔCt method. Each symbol represents the mean of 3 samples from one experiment, symbol colour marking each independent experiment. n = 2 experiments. Results from one-way RM ANOVA and Dunnett’s multiple comparison test are indicated as *: p ≤ 0.05; **: p ≤ 0.01.

To further investigate the effect of HES on GMDC activation in response to LPS, we compared gene-expression profiles of LPS-treated and LPS + HES-treated cells (Fig 3A**)**. It was noted that HES broadly reduced the transcript levels of activation markers such as costimulatory molecules (e.g., CD40 and CD83) and pro-inflammatory cytokines (e.g., IL-12p35, LT-α and IL-23p19), and even some TLRs (e.g., TLR1 and TLR6). On the other hand, HES-treatment increased the expression of type-2 markers such as IL-33 and arginase 1, and transcripts of genes involved in cellular metabolism such as HIF1A, Hmox, and Txnip (S3 Fig). Interestingly, the highest induced transcript in HES-treated GMDCs was a TGF-β-inducible gene, hinting at an influence of the TGF-β-mimic [58] in HES on DC gene expression.

To verify some of the array results, GMDCs were treated with LPS ± HES and levels of mRNAs for both subunits of IL-12, p35 (*IL12A)* and p40 (*IL12B*), as well as *TNF*, *IL6* and *Arg1* were measured by real time qPCR. As shown in Fig 3B, HES significantly reduced responses to LPS for each cytokine except TNF. *Arginase-1* transcript levels, on the other hand, were reduced after LPS stimulation, and significantly increased in the presence of HES. Taken together, HES is therefore able to modulate DC activation at the transcript level.

### The DC-modulator in HES is heat labile and can be traced to specific chromatography fractions

**Fig 4:**
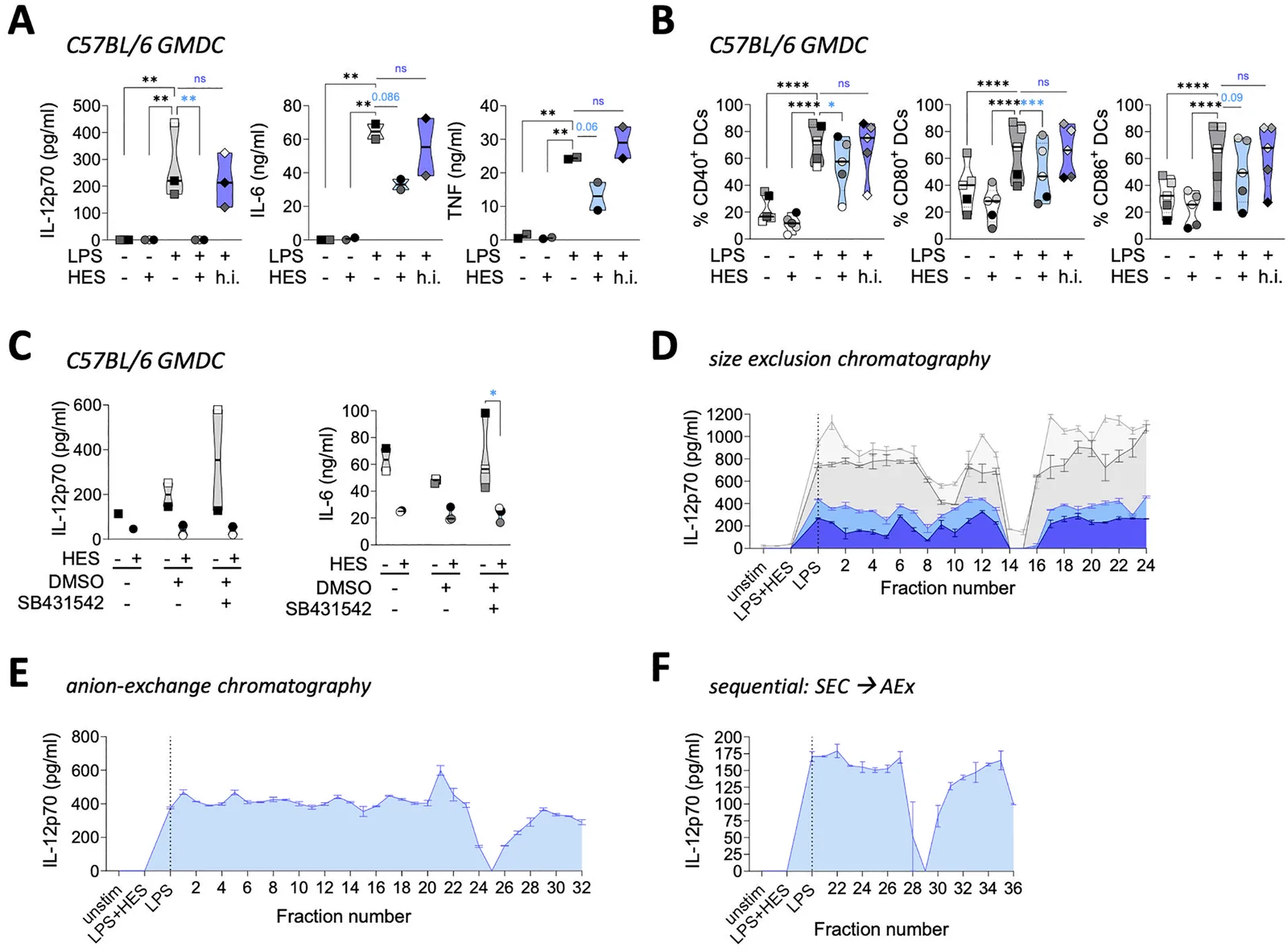
The DC-modulator in HES is heat-labile and can be traced to specific chromatography fractions. GMDC of C57BL/6 mice were differentiated with GM-CSF for 10 days before incubation with LPS and HES for 18 h. A. Secretion of IL-12p70, IL-6 and TNF by GMDC stimulated with LPS, HES and heat-inactivated HES (h.i.), measured by ELISA. B. Expression of CD40, CD80 and CD86 on CD11c^+^ cells stimulated as in A was determined by flow cytometry. C. Secretion of IL-12p70 and IL-6 by GMDC treated with DMSO or the inhibitor SB431542 before stimulation with LPS and treatment with HES, measured by ELISA. D-F. HES was fractionated by the indicated methods and then used to inhibit LPS-induced activation of GMDCs. IL-12p70 production in response to the indicated treatments was measured by ELISA. Each line represents the mean of 2 or 3 samples from one experiment. In the SEC panel, line and shading colour marking different experiments, grey and blue colouring indicates which of two different fractionation batches was tested. For A-C, each symbol represents the mean of 3 samples from one experiment, symbol colour marking each independent experiment. n = 2–5 experiments. Results from RM one-way ANOVA and Dunnett’s multiple comparisons test (A and B) or a mixed effects model with Šídák’s (C) multiple comparison test are indicated as *: p ≤ 0.05; **: p ≤ 0.01; ***: p ≤ 0.001; ****: p ≤ 0.0001. AEx: Anion-exchange chromatography, SEC: Size exclusion chromatography. unstim: untreated control.

We next aimed to identify the molecule within HES that is responsible for the modulatory effect on DC activation. Interestingly, brief heat-inactivation of HES (5 mins, 95°C) was sufficient to abolish all inhibitory activity in GMDCs stimulated with LPS (Fig 4A, 4B). This indicates that the modulatory molecule is a protein or has a necessary protein-component, which can be denatured by heat-treatment. As we have previously described several proteins in HES with TGF-β-like activity [58–60], and considering prominence of the TGF-β-inducible transcript (*Tgfbi*) in LPS + HES-treated GMDCs (Fig 3A), we tested the effects of SB431542, which inhibits the kinase activity of the type I TGFβ receptor ALK5 [61]. However, the inhibitory activity of HES remained intact in the presence of the inhibitor, excluding this possibility (Fig 4C).

We next chose to narrow down the list of potential DC modulators by fractionating HES using either size exclusion chromatography (SEC) or anion-exchange chromatography (AEx). Using two replicate SEC fractionations of HES, fractions 14 and 15 consistently inhibited IL-12p70 secretion in response to LPS-stimulation (Fig 4D). Separation by AEx resulted in a single fraction, fraction 25, displaying the activity (Fig 4E). None of these fractions contained the TGF-β-mimicking proteins previously described [58,59], or other known immunomodulators such as proteins targeting IL-33 and its receptor ST2 [62–65]. All fractions were analysed by mass spectrometry to identify proteins present in individual fractions of HES, with over 300 identified in the active fractions. We then separated the active fractions from SEC batches by AEx to produce a single active fraction (Fig 4F). A subset of 5 candidates with elution profiles matching the biological activity (S4A Fig and S1 Table) were selected for expression as recombinant proteins; however, none were found to replicate the ability of HES to block IL-12, IL-6 or TNF responses (S4B Fig).

### Pre-incubation or post-incubation of DCs with HES impairs LPS-induced activation

**Fig 5:**
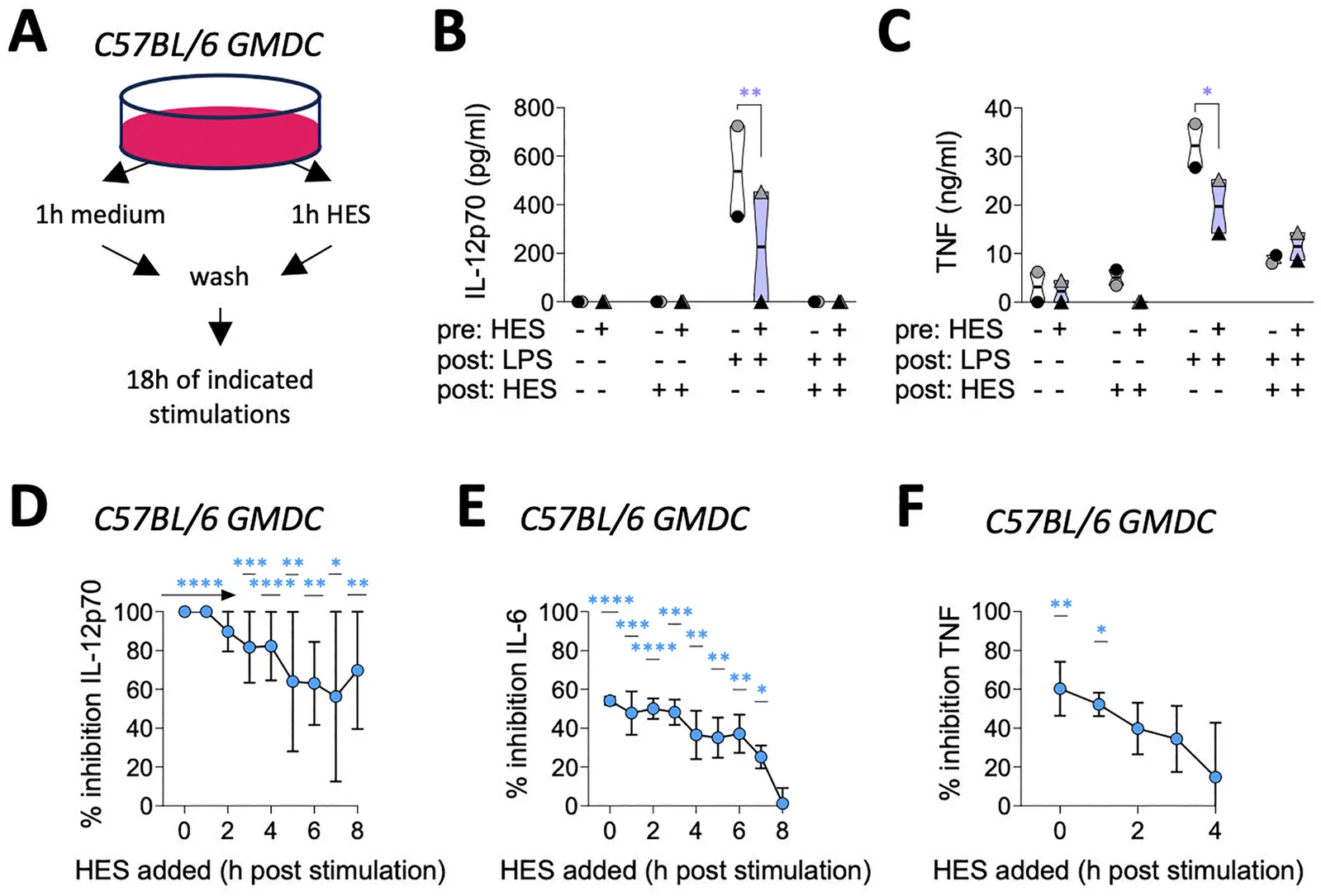
Pre-incubation or post-incubation of GMDC with HES impairs LPS-induced activation. GMDC of C57BL/6 mice were differentiated with GM-CSF for10 days before treatment as indicated. A. Schematic of the experimental setup. Day 10 GMDC were incubated with medium alone or 10 µg/mL HES in medium for 1 h at 37°C before washing and treatment with LPS and HES as indicated. B,C. Secretion of IL-12p70 (B) and TNF (C) was measured by ELISA. Samples pre-treated with HES are purple violins with triangle symbols, stimulation after the washing step is indicated below the x-axis. Each symbol represents the mean of 3 samples from a single experiment, symbol colour marking each independent experiment. n = 2 experiments. D-F. HES at 10 µg/mL was added at the indicated time points after stimulation. Cytokine concentrations in culture supernatants after 18 h stimulation were measured by ELISA and converted to percent inhibition using the LPS-treated (0% inhibition) and unstimulated cells (100% inhibition) as comparators. Data show means ± SEM of 3 averaged samples from 4 independent experiments for IL-12p70 and 3 for IL-6 and TNF. Results from 2-way RM ANOVA matching both factors and Šídák’s (B and C) multiple comparison test are indicated as *: p ≤ 0.05; **: p ≤ 0.01; ***: p ≤ 0.001; ****: p ≤ 0.0001, or mixed-effects model with Dunnett’s multiple comparison test in (D-F) for the comparison of the LPS stimulated groups (0% inhibition) to each time point. Sources of variation for the 2-way ANOVA are compiled in Supplementary Information (Statistical and Data Annex).

To explore the temporal relationship between the addition of HES and its ability to inhibit LPS-induced DC activation, we performed pre- as well as post-activation treatment experiments. First, C57BL/6 GMDCs were incubated for one hour in cRPMI with or without 10 µg/mL of HES. After a washing step, cells were then stimulated with LPS for 18 hours (see Fig 5A for a schematic of the workflow).

In replicate experiments, pre-incubation of GMDCs with HES for 1 hour prior to wash and challenge with LPS in the absence of HES significantly reduced the subsequent production of IL-12p70 (Fig 5B). Production of both TNF (Fig 5C) and IL-6 (S5A Fig) were also reduced in GMDCs pre-incubated with HES.

We next tested whether HES could inhibit cytokine responses when added later during the course of the TLR response. GMDCs were stimulated with LPS adding HES 2–8 hours later. Concentrations of IL-12p70 and TNF in the cell supernatants after 18 hours were determined, as was the expression of costimulatory molecules. Notably, IL-12p70 concentrations released by GMDCs were reduced by >50% even if HES was added up to 8 hours post-LPS stimulation (Figs 5D, S5B). The inhibition of IL-6 secretion was not quite as marked but still very distinct, with cells that received HES up to 3–6 hours post-LPS secreting barely more IL-6 than those that received both at the same time. Beyond this, IL-6 concentrations further increased the later HES was added, and there was no effect visible if HES waws added 8 hours post-stimulation (Figs 5E, S5C). The inhibition of TNF production showed a similar pattern, although it declined more rapidly, with significant inhibition no longer evident at 3 hours post-stimulation (Figs 5F, S5D).

The expression of costimulatory molecules was similarly affected. The expression of CD40 on cells that received HES 8 hours post-LPS was as subdued as in cells that had received both at the same time (S5E, S5F Fig). In contrast, the expression of CD86 seemed to increase so rapidly and robustly that HES was unable to influence it even if given only an hour post-stimulation (S5G, S5H Fig).

### The inhibitory effects of HES are independent of early signalling through ERK, p38, JNK or PI3K

To place these phenotypic and temporal patterns into context, we then investigated the status of canonical TLR signalling pathways in HES-exposed GMDCs. We started with the ERK1/2 branch of the MAPK cascade, which can modulate DC activation and drive type 2 activation [66]. GM-CSF GMDCs were stimulated with LPS with or without HES, following which ERK1/2 phosphorylation levels during the early phase of activation were analysed by flow cytometry.

**Fig 6:**
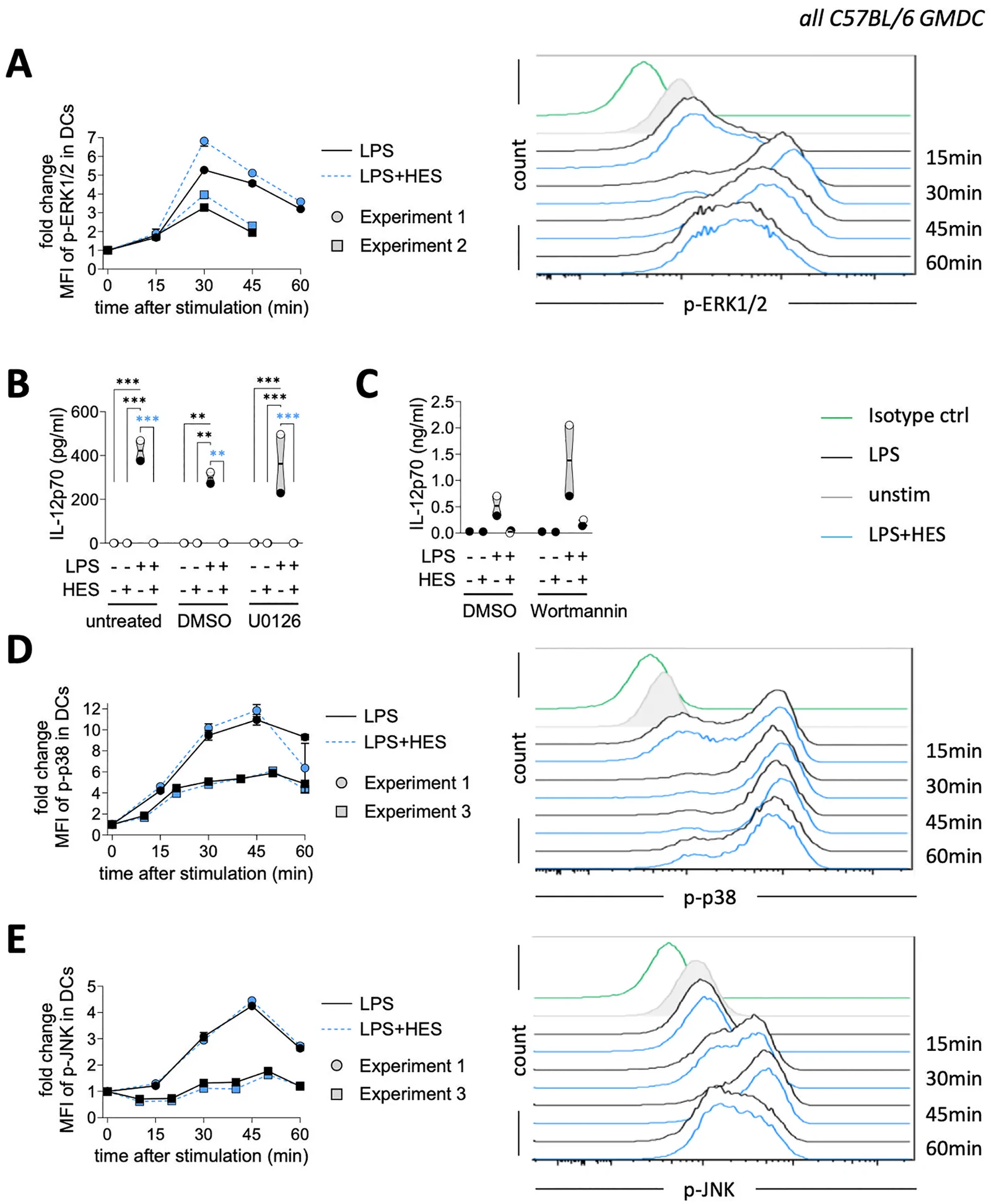
The inhibitory effects of HES are independent of early signalling through ERK, p38, JNK or PI3K. GMDC were differentiated with GM-CSF for 10 days before stimulation with LPS and HES as indicated. A. Phosphorylation status of ERK1/2 in LPS and LPS + HES treated CD11c^+^ cells was analysed by flow cytometry and quantified by determining mean fluorescence intensities (MFI), which were normalized to the unstimulated controls. Data from 2 independent experiments are shown, distinguished by the indicated symbols. B. Cells were treated with media alone or media containing DMSO or the MEK1/2 inhibitor U0126 before stimulation with LPS and HES as indicated. IL-12p70 concentrations in the supernatants were measured by ELISA. C. GMDC were treated with the PI3K inhibitor Wortmannin (100 nM) before stimulation with LPS and HES. IL-12p70 concentrations in the cell supernatants were measured by ELISA. D,E. Phosphorylation status of p38 (D) and (E) JNK in LPS and LPS+HES treated GMDC was analysed by flow cytometry, and quantified by determining MFI values, which were normalized to the unstimulated controls. Data from 2 independent experiments are shown, distinguished by the indicated symbols. Data in A, D, E show means ± SEM of 3 samples for each experiment. For data in B, each symbol represents the mean of 3 samples from one experiment, and data in C from 2 or 3 samples; symbol colour marking each independent experiment. n = 2 experiments for each panel except for some groups in C which were tested in only one experiment. In B, results from 2-way RM ANOVA and Tukey’s multiple comparison test are indicated as *: p ≤ 0.05; **: p ≤ 0.01; ***: p ≤ 0.001; ****: p ≤ 0.0001; Sources of variation for the 2-way ANOVA are compiled in Supplementary Information (Statistical and Data Annex). Black symbols mark comparison of LPS-treated cells to the unstimulated controls, blue symbols indicate the comparison of LPS and LPS + HES-treated cells at each time point.

Both treatments rapidly induced phosphorylation of ERK1/2, but levels of p-ERK1/2 in LPS + HES-treated CD11c^+^ cells at 30 mins post stimulation appeared elevated compared to LPS-alone-stimulated cells. After this early increase in phosphorylated ERK1/2, levels declined in both groups but were still slightly elevated in LPS + HES-treated compared to LPS-stimulated cells at 45 minutes and one hour after their stimulation (Fig 6A).

To ascertain whether this increase in ERK activity was relevant for the inhibition of LPS induced DC activation by HES, we tested a variety of small molecule kinase inhibitors that are widely used although not always specific for a single enzyme. First, cells were treated with an inhibitor of the MEK1/2 kinase that phosphorylates ERK1/2, namely U0126. This inhibitor completely abolished the phosphorylation of ERK1/2 induced by LPS (S6A Fig). IL-12p70 secretion by LPS-stimulated GMDCs was only slightly attenuated by pre-treatment with DMSO or U0126, while HES inhibited secretion of this cytokine irrespective of treatment (Fig 6B). Expression of the costimulatory molecules CD40 and CD80 was also inhibited by HES in the presence of U0126 (S6B, S6C Fig). The potentially increased activation of the ERK pathway is therefore dispensable for the inhibition of LPS-induced DC activation by HES.

In addition to the ERK MAPK pathway, PI3K signalling can modulate responses to TLR ligation [67–69]. We therefore pre-treated GMDC with the PI3K inhibitor wortmannin for 30 mins, before stimulation with LPS or LPS + HES. LPS stimulation induced secretion of IL-12p70 in cells pre-treated with DMSO as well as those pre-treated with wortmannin, and additional treatment with HES inhibited this IL-12p70 production in both groups (Fig 6C). The inhibitory effect of HES on DC activation is therefore independent of PI3K signalling.

Next, we investigated the activity of the other two branches of the MAPK cascade that result in phosphorylation of p38 and JNK, respectively. Again, GMDCs were stimulated with LPS and HES and then phosphorylation levels of p38 and JNK were measured by flow cytometry. Phosphorylation of both p38 and JNK rapidly increased post stimulation, peaking at 45–50 minutes after LPS-treatment. There was no difference between LPS- and LPS + HES-treated groups in this first hour (Fig 6D, 6E).

### HES treatment reduces the duration of signalling events after the immediate-early phase of GMDC activation

As treatment with HES did not affect the immediate-early phase of p38 or JNK signalling, we also evaluated levels of phosphorylated p38 over the 18 hours following LPS stimulation. We noted a tendency for p38 to decline more rapidly in LPS + HES-treated cells to compared to LPS-stimulated cells (S7A Fig). Similarly, phosphorylation of JNK declined faster in the LPS + HES-treated than in the LPS-treated group, returning to pre-stimulation levels of p-JNK after 7.5 to 10 h (S7B Fig). In addition, we probed the time-course of NF-κB signalling, as this pathway is crucial for DC activation and antigen presentation [70], measuring phosphorylation of IκBα. Again, the loss of phosphorylation over time was more pronounced in LPS + HES treated compared to LPS-stimulated DCs from both C57BL/6 (S7C Fig) and BALB/c (S7D Fig) mice. Overall, these data suggest interference with the late phase duration of signalling, reducing phosphorylation levels of p38, JNK and IκBα closer to pre-stimulation values earlier in HES + LPS-treated than in LPS-treated cells.

### HES does not influence activation induction but curtails its maintenance

To put the collected data of inhibition of MAPK and NF-κB signalling at the intermediate and late phases of activation into context regarding the actual inhibition of cytokine secretion and costimulatory molecule expression from LPS-stimulated GMDCs by HES, supernatants and cells were collected at different time points during the stimulation. Concentrations of IL-12p70, TNF and IL-6 in the supernatants and expression of costimulatory molecules were measured. In this way, a timeline could be created for the production of these markers and the onset and extent of inhibition.

**Fig 7:**
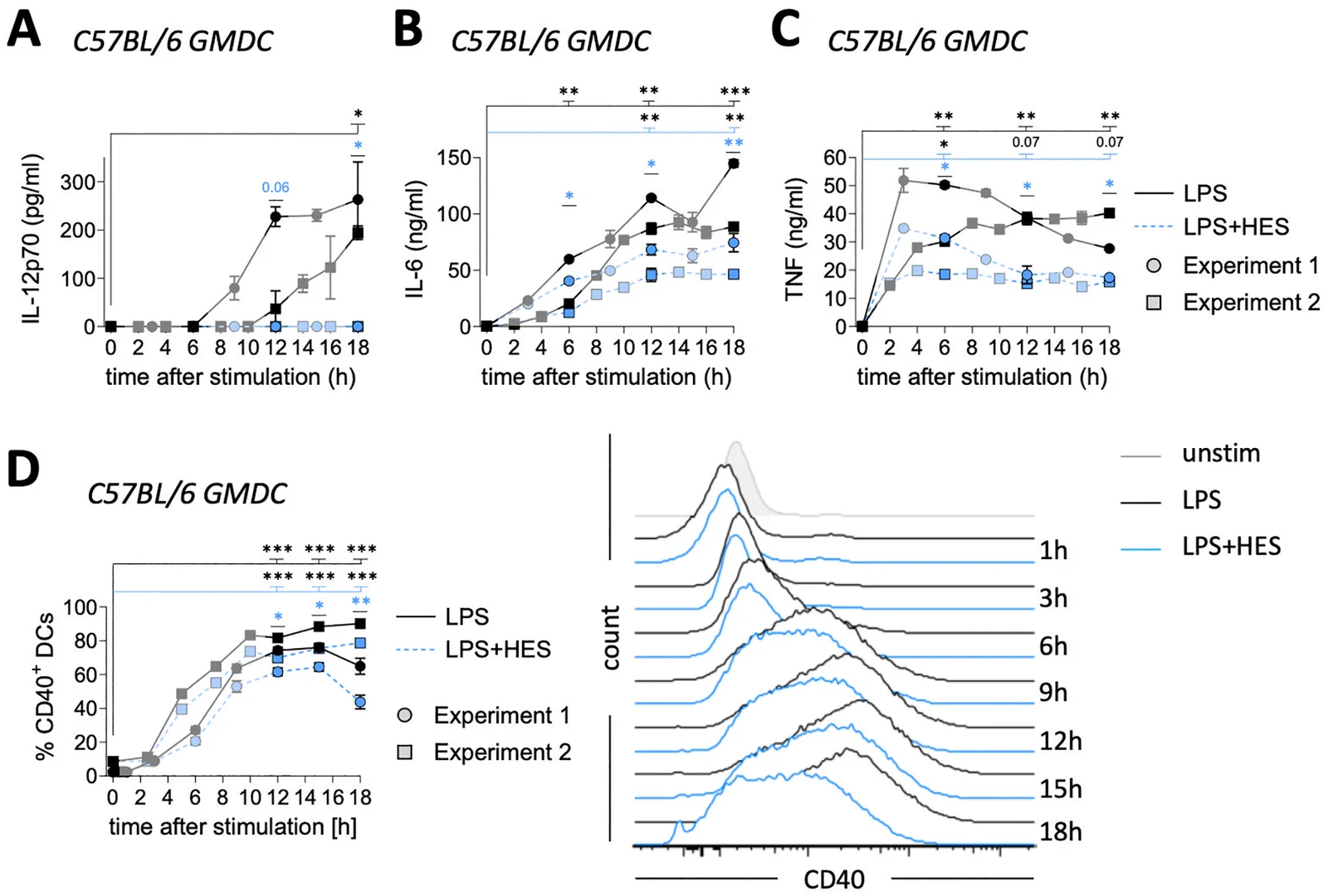
HES does not influence activation induction but curtails its maintenance. GMDC were differentiated with GM-CSF for 10 days before stimulation with LPS and HES as indicated. At indicated time points after stimulation, cells were harvested and concentrations of (A) IL-12p70, (B) IL-6, and (C) TNF in their supernatants measured by ELISA; (D) shows the percentage of CD40^+^ cells in the CD11c^+^ population at these time points. Data from 2 independent experiments are shown, distinguished by the indicated symbols. Data shown are means ± SEM of 3 samples for each experiment. Results from 2-way RM ANOVA and Dunnett’s multiple comparison test are indicated as *: p ≤ 0.05; **: p ≤ 0.01; ***: p ≤ 0.001; ****: p ≤ 0.0001. Sources of variation for the 2-way ANOVA are compiled in Supplementary Information (Statistical and Data Annex). Black symbols mark comparison of LPS-treated cells to the unstimulated controls, blue symbols indicate the comparison of LPS and LPS + HES-treated cells at each time point. Greyed out time points indicate lack of statistical analysis for these times.

LPS-stimulated GMDCs started secreting IL-12p70 quite late, between 8–12 h post stimulation. Its concentration in culture supernatants then increased over the following 4–8 h. No detectable IL-12p70 was produced by LPS + HES-treated GMDCs at any point during the stimulation (Fig 7A).

In the case of IL-6, secretion started earlier compared to IL-12p70 and was measurable in both groups, but only small amounts were detectable for the first few hours post-LPS stimulation. At 6 hours post-stimulation, LPS-treated GMDC produced significant levels of IL-6, with a noticeable inhibitory effect of the HES treatment. Until that point, LPS- and LPS + HES-treated GMDCs secreted comparable amounts of IL-6. While IL-6 concentrations increased in the supernatants of both groups in the following hours, the LPS-treated cells soon surpassed their LPS + HES-treated counterparts, which showed only very slight increases in IL-6 beyond 6 h (Fig 7B).

In contrast, the release of TNF was induced from quite early time points. Unstimulated DCs did not express any detectable amount of this cytokine, but it was detectable at two hours post stimulation of both LPS- and LPS + HES-treated GMDC. While the concentration of TNF strongly increased in GMDCs treated with LPS, in the presence of HES little further increase was observed and indeed levels trended downwards as the experiment progressed (Fig 7C).

The expression of CD40 on CD11c^+^ cells followed a similar dynamic as IL-6 secretion. While cells at three hours post stimulation had barely changed their expression of this costimulatory molecule, the percentage of CD40^+^ cells had strongly started increasing at five hours post stimulation with a trend towards higher percentages of CD40^+^ cells in the LPS-treated group already visible. In both groups the expression of this marker increased over the next hours, with HES-treated cells consistently showing lower CD40 surface levels, until expression of this marker reached a plateau after 10–12 hours in both groups (Fig 7D).

### In a transfer model of LPS-treated GMDCs, HES protects against loss of Tregs

We finally evaluated whether *in vitro* pre-treatment of GMDCs with HES altered the outcome when cells were adoptively transferred to the *in vivo* setting, using a model in which DCs are delivered subcutaneously in the ear pinna, and cells collected from the draining cervical lymph node 24–96 hours later; in this model, the contralateral ear is injected with PBS and the lymph node compared to that draining the ear receiving GMDCs. We first established that significant numbers of DCs migrated to the cervical lymph nodes within 24 hrs (S8A, S8B Fig); HES treatment appeared to increase the total numbers of DCs, so that no inhibition of migration was evident. In additional experiments, we analysed T cell subsets 96 hrs post-injection with LPS or LPS + HES GMDCs; although Tbet^+^ Th1 cells were similar in the two groups, only in LPS-treated and not LPS + HES-treated animals was a significant reduction of Foxp3 ^+^ Tregs observed (S8C, S8D Fig).

## Discussion

Dendritic cells are pivotal in forming an appropriate immune response to helminths, but the parasites have evolved multiple strategies to defuse and divert DC-driven immunity [11,48]. Here we detail the effects of HES, from the intestinal nematode *H. polygyrus*, on the process of DC activation. Multiple studies over a number of years have reported that expression of IL-12 in DCs stimulated with the TLR ligand LPS is inhibited by products from many helminth species, including *Ascaris suum* [71,72], *Echinococcus granulosus* [69], *Mesocestoides corti* [73], *Nippostrongylus brasiliensis* [36], *Schistosoma mansoni* [74,75], *Trichinella spiralis* [75–77] and *Trichuris suis* [75], as well as HES from *H. polygyrus* [37]. Inevitably, these studies involved a variety of DC types from mouse or human, differentiated under heterologous conditions with varying definitions of the resulting DC subsets. There is also a paucity of information on the broader set of response phenotypes across TLR ligands, and the downstream events following helminth perturbation of these responses. To pursue these questions, we selected HES as one of the most closely studied helminth products with known immunomodulatory activity in vitro and in vivo [48,78].

The first point we addressed was whether HES specifically inhibits DC activation by certain stimuli but not others. To this end, GMDCs were stimulated with ligands for TLR1/2, 3, 4, 7 and 9 and the effect of concomitant treatment with HES on the expression of costimulatory molecules and secretion of pro-inflammatory cytokines analysed. As expected after the above-mentioned reports, HES was found to inhibit DC activation after LPS and CpG stimulation. In addition, it was also able to down-modulate activation induced with the three other TLR ligands, in both C57BL/6 as well as BALB/c GMDCs. Direct interference with the TLR ligands themselves is unlikely as pre-exposure of cells to HES, followed by washing and administration of ligands, maintained the inhibited phenotype.

Furthermore, it was clear that HES could impact the intracellular protein levels of at least some of the regulated proteins, as the percentage of IL-12^+^ GMDCs decreased in LPS-stimulated samples upon addition of HES. Even more interesting, this reduction could also be observed measuring the levels of the mRNAs for the IL-12 subunits and other LPS-induced inflammatory mediators such as IL-6 at eight hours after stimulation. It is possible this indicates an effect of HES on RNA stability, similar to the mechanism of action of the ribonuclease Omega-1 that was found in SEA [29]. However, we did not observe any changes in RNA quantity or quality after HES treatment, and we could detect transcripts that were in fact increased even compared to unstimulated cells, such as *Arg1*. This speaks to a broader modulatory effect on the DC transcriptome, with subsequent changes in DC activation.

Most studies reported to date have been performed in GM-CSF differentiated GMDCs. While a separate macrophage-like population in these cultures responds in a distinct way [53] we showed similar inhibition in CD115-depleted cell populations. An alternative method of generating DCs from mouse bone marrow uses Flt3-L as differentiation factor. With this method three populations of FLDCs are created, which correspond to murine splenic DC populations: CD11c^+^ B220^+^ cells (pDCs) and two populations of CD11c^+^ B220^−^cDCs (CD24^+^ cells corresponding to CD8^+^ cDC1s and CD11b^+^ cells corresponding to CD11b^+^ cDC2s) [57]. Upon treatment with HES, LPS-induced maturation of all three of these subsets was inhibited, confirming that HES is a broadly-acting modulator of DC responses.

We next aimed to pinpoint the target of HES in the DC activation process. Since activation through various TLRs was inhibited, we hypothesised that HES might target downstream signalling pathways shared between these receptors, such as the MAPK cascade. The first MAP kinase investigated here was ERK. HES increased the phosphorylation of ERK1/2 during the first half hour after GMDC stimulation with LPS. This finding was not surprising considering work by Agrawal et al., showing that in human moDC, the induction of ERK1/2 phosphorylation by the TLR2 ligand Pam3cys results in an inhibition of IL-12p70 production which is restored upon inhibition of ERK1/2 activation [66]. It was therefore an interesting possibility that HES inhibits DC activation via increased activation of ERK1/2. However, HES was still able to inhibit DC activation when cells were treated with an inhibitor of MEK1/2, the kinases that phosphorylate ERK1/2, demonstrating that this is not its mechanism of action.

In contrast to ERK1/2, both p38 and JNK have been shown to be important for the maturation of DCs. Inhibition of either MAP kinase resulted in a reduced upregulation of costimulatory molecules and impaired expression of IL-12p70 and TNF by human moDCs after LPS stimulation [66,79]. Here, these two branches of the MAPK cascade were not affected in the first hour post-stimulation, as phosphorylation of p38 and JNK was comparable in GMDCs stimulated with LPS and LPS + HES. When looking at later time points however, it became clear that HES did have an effect on these signalling pathways, as in cells treated with HES the phosphorylation levels of both p38 and JNK returned to pre-stimulation levels more quickly than in LPS-treated cells. Another signalling node in DC activation is NF-κB. Investigation of NF-κB signalling via analysing the phosphorylation status of IκBα led to a similar result as with the MAPK pathways. While we did not investigate the early time points after activation, HES did seem to induce an earlier and more profound loss of IκBα phosphorylation. Future studies to explore this question may also investigate whether HES interferes with downstream events following transcription factor phosphorylation, for example by blocking nuclear translocation or interactions with essential partner proteins required for the classical TLR response.

These results hinted to an effect of HES not on the initial early phase of activation but on the ability of DCs to sustain the LPS-induced activation. This was further supported by the data gathered from expression time course experiments with IL-12, TNF, IL-6 and CD40. Those of these proteins that were induced early on, which were IL-6, CD40 and especially TNF, were induced to the same extent in the first three to four hours of stimulation. From that time point, HES exerted different effects on each protein. While it blocked any further increase in secreted TNF, levels of IL-6 and CD40 continued rising, albeit to a lesser extent than in cells stimulated with LPS alone. IL-12p70 on the other hand, which was detectable in the cell supernatants only at a much later time than the other two cytokines, was immediately suppressed by HES. Such a delayed action indicates that HES altered the transcriptional and metabolic profile of DCs, supported by gene expression analysis and quantification of inflammatory cytokine mRNA transcripts.

The hypothesis that HES inhibits the maintenance rather than the induction of DC activation is further supported by the results showing its ability to impact LPS-induced secretion of IL-12p70, IL-6, and TNF, and the upregulation of CD40 even if added hours after the start of the stimulation. In fact, we found that the effect of HES on TNF started to wane if administration was delayed until three to four hours of LPS stimulation, which was around the same time that protein levels in the expression time course start showing the inhibitory effect of HES.

The results so far indicate an impressive ability of HES to suppress DC activation in a manner that would be antigen-independent, but in being slow-acting may also be long-lasting, particularly in the context of a chronic infection with continuous exposure to parasite products, thereby forestalling an effective response that would expel the parasites. Although delayed, such effects on DCs represent a very early stage of a long-term infection. It could also be argued that the profound inhibition of DC activation to bystander antigens may be a mechanism developed by the parasite to protect the host from a potentially deadly response to the intestinal microflora that ensues after *H. polygyrus* breaching the intestinal wall. In either setting, TLR activation *in vivo* will not be a discrete event but occurs in a continuum, punctuated by worm development and migration, which can then result in bacterial translocation. Hence on all accounts, interference with DC-initiated inflammation is likely to be critical for worm survival.

One objective of the study was not attained: to identify the active molecule in HES that modulates DC function. While our strategy was comprehensive, employing both size-exclusion and anion exchange chromatography, and detailed mass spectrometry to identify candidates with an elution profile matching the biological activity in the chromatographic fractions, none of the selected proteins recapitulated the inhibition shown by HES. In this work, we expressed recombinant proteins in mammalian cells (in part to exclude any bacterial contamination when testing on DCs), but these would not carry the post-translational modifications (PTMs) of the native parasite product. This discrepancy is not unprecedented, as both of the most well-characterised helminth DC-modulatory molecules require the addition of N-glycosylated side chains for biological activity: in the case of *A. viteae* ES-62, the protein bears immunoactive phosphorylcholine [16], while *S. mansoni* Omega-1 is conjugated with Lewis-X [80] that mediates DC uptake via the mannose receptor [29]. Similarly, products of *T. suis* that modulate human DCs rely on glycan interactions with cell surface lectin receptors [34]. As all but one of the 5 candidates we identified in HES include potential N- or O-glycosylation sites (S1 Table), and the glycans on HES constituents have been described [29], future studies should investigate whether PTMs are involved in HES-DC interactions.

In conclusion, we demonstrate a broad modulatory effect of HES on DC activation, which does not impact the early stages of the activation process and instead cuts short signalling events after the initial activation stimulus. In the context of an enduring infection that establishes for weeks or months in the host, the ability to interfere with later downstream events may provide a more robust and pro-homeostatic strategy for parasite down-modulation of inflammation consistent with its evolutionary adaptation for survival in the infected host.

## Materials and methods

### Ethics statement

For studies on human monocyte-derived dendritic cells (moDCs), apheresis cones were sourced from healthy donors and acquired from the NHS blood service under ethics license 2017-2551-3945 approved by the University Research Ethics Committee (UREC) of the University of Manchester.

### Animals

For this work, both male and female C57BL/6 and BALB/c mice aged 6–8 weeks, as well as male and female OT-II mice 8–16 weeks of age were used. These were bred in-house at The University of Edinburgh and the University of Glasgow, and maintained under specific pathogen-free conditions. All work was performed under licenses granted by the Home Office (UK), in accordance with local guidelines.

### Cell isolation and culture

Bone marrow was extracted from femurs and tibiae of mouse hind legs. To generate GMDCs, single cell suspensions were plated at 2x10^6^ cells/mL in cRPMI (10% FCS, 2 mM L‐glutamine, 50 U/mL penicillin, and 50 μg/mL streptomycin) plus 20 ng/mL GM-CSF (PeproTech). An additional 10 mL of GM-CSF-containing medium was added on day three and exchanged on days 6 and 8 of culture at 37°C with 5% CO_2_. To generate FLDCs [57], red blood cells were lysed (Sigma RBC lysis buffer, 5 mL, 4min) and incubated at 1.5x10^6^ cells/mL in cRPMI with 200 ng/mL Flt3L for eight days. Splenocytes were isolated by maceration through 70 µm nylon cell strainers, red blood cell lysed (Sigma RBC lysis buffer, 5 min) and enriched for CD11c^+^ cells using biotinylated anti-CD11c (clone N418, eBioscience) and streptavidin-beads on MACS separation columns (Miltenyi Biotec) according to the manufacturer’s protocol. To generate human moDCs, peripheral blood mononuclear cells were isolated from apheresis cones collected at a blood bank via density gradient centrifugation (40 min, 400 *g*), followed by enrichment for CD14^+^ cells by MACS (Miltenyi Biotec); cells were seeded at 0.5x10^6^ cells/mL in cRPMI with 25 ng/mL IL-4 and GM-CSF and matured for 6 days with a medium exchange on day three.

### DC stimulation and inhibition

Murine GMDCs were stimulated at 10^6^ cells/mL with 100 ng/mL Pam3CSK4 (Invivogen), 580 µg/mL Poly(I:C), 1 µg/mL LPS (Sigma), 1 µg/mL R848 (Invivogen) or 10 µg/mL CpG (Invivogen) in cRPMI, with 5 ng/mL GM-CSF. Murine FLDCs were stimulated at 2x10^6^ cells/mL with 100 ng/mL LPS (Sigma) and 50 ng/mL Flt3L in cRPMI. Human moDCs were stimulated at 0.5x10^6^ cells/mL in cRPMI with 25 ng/mL IL-4 and GM-CSF and 100 ng/ml LPS. HES, prepared as described previously [81] and checked for its LPS content by LAL assay (Lonza,  < 1.0 Endotoxin Units/µg), was used at 10 µg/mL unless indicated otherwise, and added to cultures as described in text and figure legends. To inhibit MEK1/2, cells were incubated with 10µM U0126 (Cell Signaling Technology) for 1 h before stimulation, while 100 nM wortmannin (Calbiochem) was used for 30 min to inhibit PI3K and 5 µM SB431542 (Tocris Bioscience) for 1 h to inhibit TGF-β signalling; DMSO was used as a control.

### Adoptive transfer of GMDCs to ear pinna

GMDCs were grown as above before stimulating with 10 μg/mL OVA, 100 ng/mL LPS, 10 μg/mL HES for 3 h. In some experiments, cells were labelled with 5 μM CSFE cell proliferation kit (Thermo Fisher) for 20 min prior to washing and resuspending cells in sterile PBS. OT-II CD45.1 mice were injected 1x10^6^ BMDCs in 50 μL into the ear pinna, the contralateral ear receiving an equivalent volume of sterile PBS. The draining cervical lymph node was dissected at either 1 or 4 days post injection and digested in 1 mg/mL collagenase D, 37°C, 250 rpm, 40 min and mashed for single cell suspension for staining for flow cytometry with antibodies as detailed below.

### HES fractionations and mass spectrometry

HES was fractionated using two different methods, size exclusion chromatography (SEC) and anion-exchange chromatography (AEX), on an ÄKTApurifier (GE Healthcare). For SEC, PBS with a flow rate of 0.5 mL/min was used over a Superdex 200 10/300 GL column to collect 500 µL fractions. For AEX, HES or active SEC fractions were dialysed into starting buffer (20 mM Tris-HCl in dH_2_O, pH 8) and then run over a Mono Q 5/50 GL column with an elution buffer gradient (20 mM Tris-HCl and 1 M NaCl in dH_2_O, pH 8; zero to 50% over 12.5 column volumes, 50–100% over 5 column volumes and 100% for 5 column volumes), a flow rate of 2 mL/min and collected fractions of 1 mL for HES and 0.5 mL for sequential fractions.

Mass spectrometric analyses of HES fractions were performed at Synthsys, The University of Edinburgh. For each fraction, 5µg were trypsin-digested, analyzed by LC MS/MS (Agilent 1200 HPLC system, Agilent, coupled to an Orbitrap XL mass spectrometer, Thermo Scientific), and compared to an in-house *H. polygyrus* transcriptomics database using Mascot, with a minimum cutoff score of 20 and a significance threshold of p = 0.05. The exponentially modified protein abundance index (emPAI) was used to estimate protein abundance in each fraction.

### Cloning and expression of recombinant proteins

Using our *H. polygyrus* database, the relevant protein sequences were identified, optimized for expression in HEK-293 cells and subsequent purification (using the ExPASy Translate tool, SignalP 4.1, the GeneArt GeneOptimizer tool, optimizing for expression in human cells and adding restriction sites and 6-His tags) and synthesized by GeneArt. Using the inserted *Asc*I and *Xho*I restriction sites, the sequences were cloned into the pSecTag2a expression vector (restriction enzymes from New England Biolabs, vector and ligation reagents from Thermo Fisher), and used to transform JM109 cells. After sequence confirmation by Sanger sequencing, the selected plasmids were extracted using the PureLink HiPure midiprep kit (Thermo Fisher) and used to transfect HEK293T cells with the calcium phosphate transfection technique [82]; 10 mL cells at 20% confluency were transfected over night with 1 mL transfection solution containing 20 µg plasmid DNA, and stable cell lines expressing the plasmids of interest were selected using HEK cell medium (DMEM with 10% FCS, 2 mM L-glutamine and 1 µg/mL Penicillin/Streptomycin) containing 100 µg/mL Zeocin (Thermo Fisher) for three to four weeks. For recombinant protein production, transfected cells were grown in serum free medium (293 SFM II media, Thermo Fisher); supernatants containing the proteins of interest were dialysed (SnakeSkin Dialysis Tubing with 10 kDa MWCO, Thermo Fisher) for purification by nickel affinity chromatography using 1 mL HiTrap Chelating HP columns (GE Healthcare) on the ÄKTApurifier (GE Healthcare) with an elution on an imidazole gradient over 20 column volumes, collecting 1 mL fractions. Fractions containing the expressed protein were pooled and dialysed into PBS using Slide-A-Lyzer Dialysis Cassettes (Thermo Fisher); protein concentrations were subsequently determined by BCA (Thermo Fisher).

### Cytokine measurements

Concentrations of IL-6, IL-12p70 and TNF in mouse cell supernatants were measured by ELISA, using anti-IL-6 (MP5-20F3, BD Biosciences) with biotinylated anti-IL-6 (MP5-32C11, BD Biosciences), anti-IL12p70 (BD Biosciences) with biotinylated anti-IL-12p40/70 (C17.8, BD Biosciences), and anti-TNF (TN3–19, eBioscience) with biotinylated anti-TNF (polyclonal, eBioscience) in standard ELISA protocols. Briefly, Nunc MaxiSorp ELISA plates were coated overnight at 4°C with the listed unlabelled antibody in bicarbonate/carbonate coating buffer (pH 9.6). After 2 h blocking with TBS containing 0.05% Tween 20 and 10% FCS at 37°C, incubation of samples and standards was performed at 4°C overnight. Bound cytokines were detected by sequential incubation with the listed biotinylated antibodies, extravidin alkaline phosphatase and SIGMAFAST p-Nitrophenyl phosphate solution made from tablets (Sigma), read on an Emax precision microplate reader (Molecular Devices). Concentrations were calculated using the SoftMax Pro software (Molecular Devices). Concentrations of cytokines produced by human moDC were measured by CBA (BD Bioscience) according to manufacturer’s recommendations, using the BD FACS Verse for detection.

### Flow cytometry

Staining was performed in 96-well plates, using the LIVE/DEAD Fixable Aqua Dead Cell Stain Kit (life technologies) for 15 min (FLDC and moDC), followed by blocking with unlabeled polyclonal IgG for 10 min and surface staining for 20 min, all at 4°C. Cells were then directly acquired on a flow cytometer. For intracellular staining, 2% formaldehyde (final concentration) was directly added into the stimulation wells at the appropriate time points to preserve phosphorylation status of signalling molecules, followed by 10min incubation at room temperature. Cells were then transferred into pre-warmed phosflow fix/lyse buffer (BD Biosciences) for a further 10 min at 37°C, before 15 min incubation in BD phosflow perm buffer at room temperature. Cells were then stained overnight at 4°C. The following antibodies were used: B220 (PerCP, clone RA3-6B2, BioLegend), CD3 (APC Biolegend, PEcy5 BD Pharmingen, all clone 17A2), CD4 (BV650, clone RM4, Biolegend), CD11b (Biotin, FITC, PB, all clone M1/70, BioLegend), CD11c (Biotin, eFluor 450, both eBioscience, and APC, BioLegend, all clone N418), CD24 (PE-Cy7, clone M1/69, BioLegend), CD19 (BV605, clone ID3, Biolegend), CD40 (PE, clone 3/23, BD Biosciences), CD44 (AF700, clone IM7, Biolegend), CD45 (BUV805, clone 30-F11, eBioscience), CD64 (BV711, clone X54-5/7.1, Biolegend), CD80 (APC, clone 16-10A1, BioLegend), CD86 (AF488 or BV650, clone GL-1, BioLegend), Foxp3 (BV421, FJK-16S, Invitrogen), Gata3 (AF488, clone 16E10A23, Biolegend), IL-12p40 (APC, clone C15.6, BD Biosciences), MHC-II (APCcy7, clone M5/114.15.2, Biolegend), p-ERK (Thr202/Tyr204; clone C13.14.4E, Cell Signaling Technology), p-IκBα (S32/S36; eF660, clone RILYB3R, eBioscience), p-JNK (T183/Y185; 81E11, Cell Signaling Technology), p-p38 (T180/Y182; D3F9, Cell Signaling Technology), RORγT (PE, clone AFKJS-9, Invitrogen), Tbet (PEcy5.5, clone eBio4B10, eBioscience),. Unlabelled p-ERK, p-p38 and p-JNK were then further stained with Zenon AF488 rabbit IgG labeling reagent (Life Technologies). Instruments used for acquisition were the FACS Canto II, LSR II or Fortessa (all BD). Data was analysed using FlowJo v9 (FlowJo LLC).

### RNA extraction and qRT-PCR

To extract RNA, GMDCs were harvested eight hours post-stimulation and resuspended in 1mL Trizol. After addition of 200 µL chloroform, samples were vortexed for 15s, centrifuged (13,000 *g,* 15 min, 4°C), and the aqueous phase mixed with 500 µL isopropanol. The mix was incubated for 10 min, followed by pelleting of RNA at 13,000 *g*, 4°C, for 10 min, one wash with 1ml ethanol and drying of the extracted RNA before resuspension in 30 µL RNase-free water.

RNA concentrations were then measured using a NanoDrop 2000 (Thermo Scientific), and quality assessed by gel electrophoresis. For the reverse transcription reaction, 1 µL oligo-dT Primer was added to 1 µg RNA in 16 mL RNase free water and incubated for 10 min at 70°C, followed by addition of 8 µL of a master mix containing 5x M-MLV reaction buffer, dNTPs, RNasin ribonuclease inhibitor and M-MLV reverse transcriptase (all reagents from Promega), and incubation for 60 min at 42°C followed by another 10 min at 70°C.

Quantitative real-time PCR was performed in duplicate per sample using the LightCycler 480 DNA SYBR Green I Master kit according to manufacturer’s recommendation, on a LightCycler 480 II (Roche). Relative expression was calculated using the ΔΔCt method; Rpl13a was used as the housekeeping gene and data was normalized to the unstimulated cells as the control group.

For the microarray analysis of gene expression changes, cDNA was biotinylated using the Illumina TotalPrep RNA Amplification Kit (Life Technologies) according to the manufacturer’s instructions. The microarray was then analysed at the Edinburgh Clinical Research Facility (WTCRF, Western General Hospital, Edinburgh), using the MouseWG-6 v2.0 Expression BeadChip (Illumina). Original data from the array analysis have been deposited with GEO under the reference GSE313792.

### Statistical analyses

Statistical analyses were performed using GraphPad Prism version 10. Unless otherwise specified, three samples per experiment were averaged and mean values analysed by 2-way or one way repeat measures (RM) ANOVA or mixed effects model, using Šídák’s or Dunnett’s multiple comparison test as appropriate. Significance levels are indicated as *: p ≤ 0.05; **: p ≤ 0.01; ***: p ≤ 0.001; ****: p ≤ 0.0001, and sources of variation for the 2-way ANOVA are compiled in Supplementary Information (Statistical and Data Annex).

Raw data from the microarray analysis of gene expression profiles from LPS and LPS + HES-treated GMDCs was quality controlled (arrayQualityMetrics Bioconductor package), the arrays normalized (robust spline normalization method after data transformation using a variance stabilizing method) and array features annotated. Fold changes were determined by group-wise comparisons and significance values for each fold change controlled for false discovery, adjusted p values of 0.05 or below have been defined as significant.

### Financial disclosure

We thank the Darwin Trust of Edinburgh for studentship support for AMK, the Wellcome Trust for funding through Investigator Awards to RMM (Ref 106122 and 219530) and the Wellcome Centre for Integrative Parasitology at the University of Glasgow (Ref: 104111). The funders had no role in study design, data collection and analysis, decision to publish, or preparation of the manuscript.

## Supporting information

S1 Fig HES inhibits activation of DCs in response to TLR ligation. (PDF)

S2 Fig HES broadly inhibits activation of different DC subsets in response to TLR ligation. (PDF)

S3 Fig HES induces a subtle but distinctive change in GMDC gene expression. (PDF)

S4 Fig The DC-modulator in HES is heat-labile and can be traced to specific chromatography fractions. (PDF)

S5 Fig Pre-incubation or post-incubation of GMDC with HES impairs LPS-induced activation. (PDF)

S6 Fig The inhibitory effects of HES are independent of early signalling through ERK, p38, JNK or PI3K. (PDF)

S7 Fig HES treatment curtails the later stages of signalling. (PDF)

S8 Fig HES-treated DCs mitigate reduction in Foxp3 + Tregs caused byLPS-treated DCs. (PDF)

S1 Table Candidate proteins tested for inhibition of DC function. (PDF)

S1 Data Original data and statistical values for Figures presented. (XLSX)

## Abbreviations

AEx: anion-exchange chromatography
BMDCs: bone marrow-derived dendritic cells (murine)
FLDCs: differentiated bone marrow-derived dendritic cells
Flt3-L: FMS-like tyrosine kinase 3 ligand
GMDCs: GM-CSF-differentiated bone marrow-derived dendritic cells
LPS: lipopolysaccharide
JNK: c-Jun N-terminal kinase
MAPK: mitogen activated protein kinase
MFI: Mean Fluorescence Intensity
MoDCs: monocyte-derived dendritic cells (human)
NF-κB: Nuclear Factor κB
PAMP: pathogen associated molecular pattern
SEC: size exclusion chromatography
TLR: Toll-like receptor
