# Supplementary material for: Curtailment of Toll-like receptor signalling and cytokine production in dendritic cells by secreted products of Heligmosomoides polygyrus
Source: PLoS Pathog. 2026 Aug 26;22(8):e1014528. doi: 10.1371/journal.ppat.1014528 (PMC13577464; doi:10.1371/journal.ppat.1014528)
Supplement: Supplementary file 1 [file ppat.1014528.s001.pdf]

Figure S1

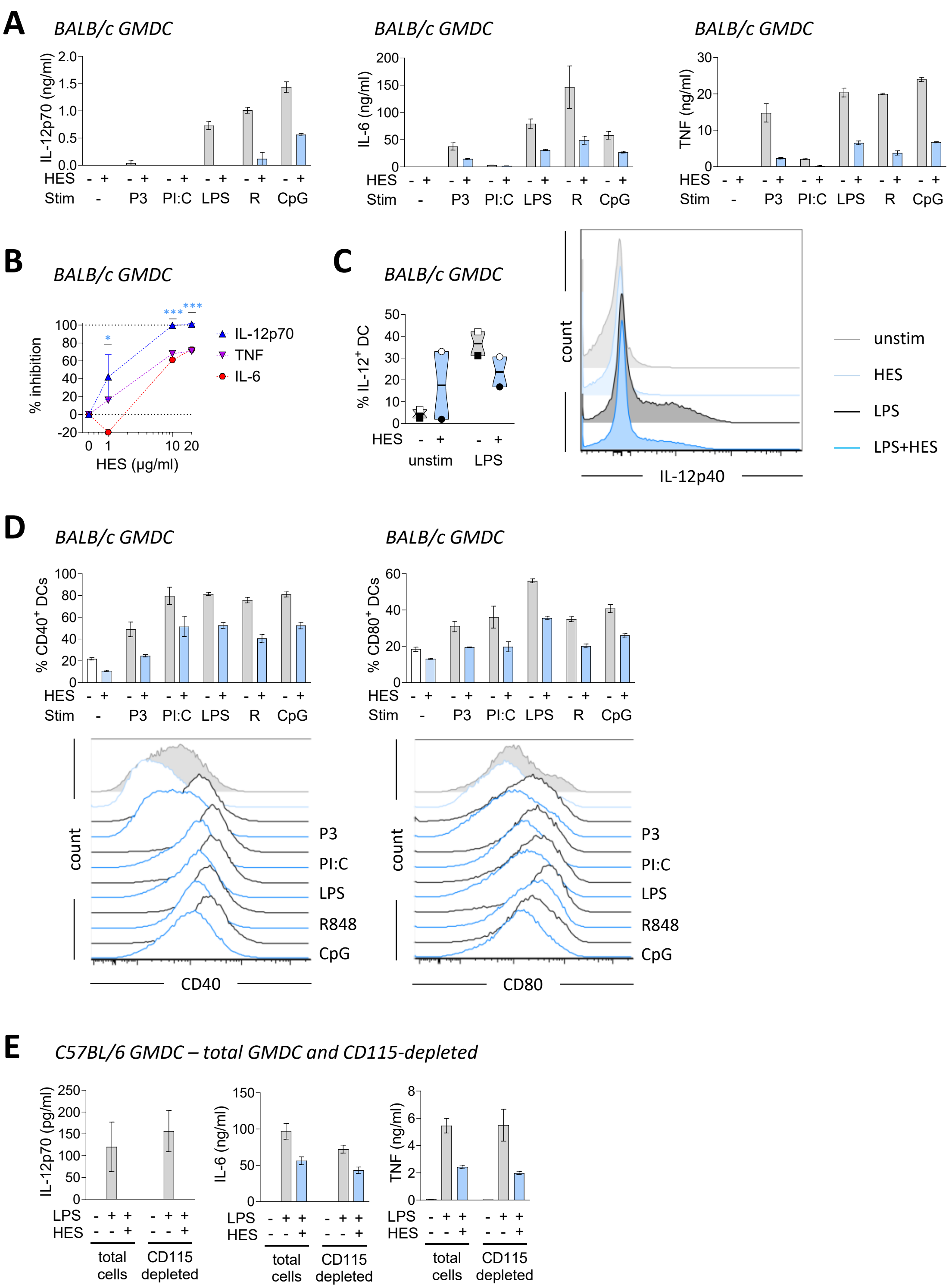
A
BALB/c GMDC
IL-12p70 (ng/ml)
IL-6 (ng/ml)
TNF (ng/ml)
HES
Stim
P3
PI:C
LPS
R
CpG
B
BALB/c GMDC
% inhibition
IL-12p70
TNF
IL-6
HES (µg/ml)
C
BALB/c GMDC
% IL-12+ DC
unstim
LPS
count
HES
LPS+HES
IL-12p40
D
BALB/c GMDC
% CD40+ DCs
% CD80+ DCs
P3
PI:C
LPS
R848
CpG
CD40
CD80
E
C57BL/6 GMDC – total GMDC and CD115-depleted
IL-12p70 (pg/ml)
IL-6 (ng/ml)
TNF (ng/ml)
LPS
HES
total cells
CD115 depleted

Suppl. Figure 1: HES inhibits activation of DCs in response to TLR ligation.

GMDC of BALB/c mice were differentiated with GM-CSF for 10 days before incubation with TLR ligands and HES for 18 h.

A. Secretion of IL-12p70, IL-6 and TNF by GMDC from BALB/c mice stimulated as indicated was measured by ELISA.

B. Dose titration of HES in GMDC from BALB/c mice. Shown are percent inhibition values of secreted IL-12p70, IL-6 and TNF , measured by ELISA . LPS-stimulated cells without HES served as 0% inhibition control, and unstimulated cells as 100%.

C. Percentage of GMDC from BALB/c mice positive for intracellular IL-12 as measured by flow cytometry. Each circle represents the mean of 3 samples from one experiment, circle colour marking each independent experiment. n = 2 experiments.

D. Expression of CD40 and CD80 on $CD11c^+$ GMDC from BALB/c mice was determined by flow cytometry. Shown are average values from 3 samples from one experiment.

E. GM-CSF-differentiated GMDC from C57BL/6 mice were either stimulated as above, or depleted of $CD115^+$ cells by MACS before stimulation. Secretion of IL-12p70, IL-6 and TNF by total GM-CSF GMDC and by CD115-depleted GMDC was measured by ELISA.

In B (for IL-12p70) and C, results from mixed-effects analysis with Dunnett's (B) or 2-way RM ANOVA and Šídák's (C) multiple comparison test are indicated as * : $p \leq 0.05$; ** : $p \leq 0.01$; *** : $p \leq 0.001$; **** : $p \leq 0.0001$. Bars in A, D and E represent the means ± SEM of 3 samples from one experiment performed.
