# Supplementary material for: Curtailment of Toll-like receptor signalling and cytokine production in dendritic cells by secreted products of Heligmosomoides polygyrus
Source: PLoS Pathog. 2026 Aug 26;22(8):e1014528. doi: 10.1371/journal.ppat.1014528 (PMC13577464; doi:10.1371/journal.ppat.1014528)
Supplement: Supplementary file 2 [file ppat.1014528.s002.pdf]

Figure S2

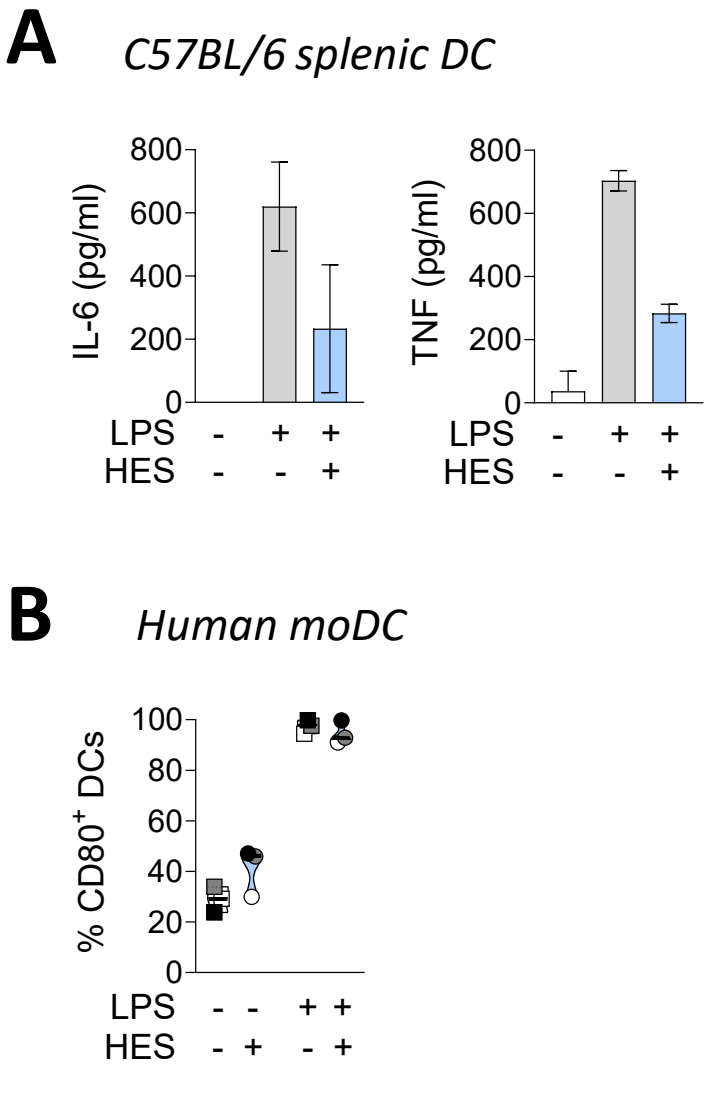

A
C57BL/6 splenic DC
IL-6 (pg/ml)
800
600
400
200
0
LPS - + +
HES - - +
TNF (pg/ml)
800
600
400
200
0
LPS - + +
HES - - +
B
Human moDC
% CD80+ DCs
100
80
60
40
20
0
LPS - - + +
HES - + - +

Suppl. Figure 2: HES broadly inhibits activation of different DC subsets in response to TLR ligation.

A. Splenocytes of C57BL/6 mice were enriched for $CD11c^+$ cells by MACS and incubated with LPS and HES for 18 h. The secretion of TNF and IL-6 was measured by ELISA. Bars represent the means ± SEM of 3 samples from one experiment performed.

B. Expression of CD80 on $CD11c^+CD14^-$ cells from samples in **Fig. 2C** was measured by flow cytometry. Each circle represents the mean of 2 samples from one donor, circle colour marking each donor. n = 3 donors.
