# Supplementary material for: Curtailment of Toll-like receptor signalling and cytokine production in dendritic cells by secreted products of Heligmosomoides polygyrus
Source: PLoS Pathog. 2026 Aug 26;22(8):e1014528. doi: 10.1371/journal.ppat.1014528 (PMC13577464; doi:10.1371/journal.ppat.1014528)
Supplement: Supplementary file 3 [file ppat.1014528.s003.pdf]

Figure S3

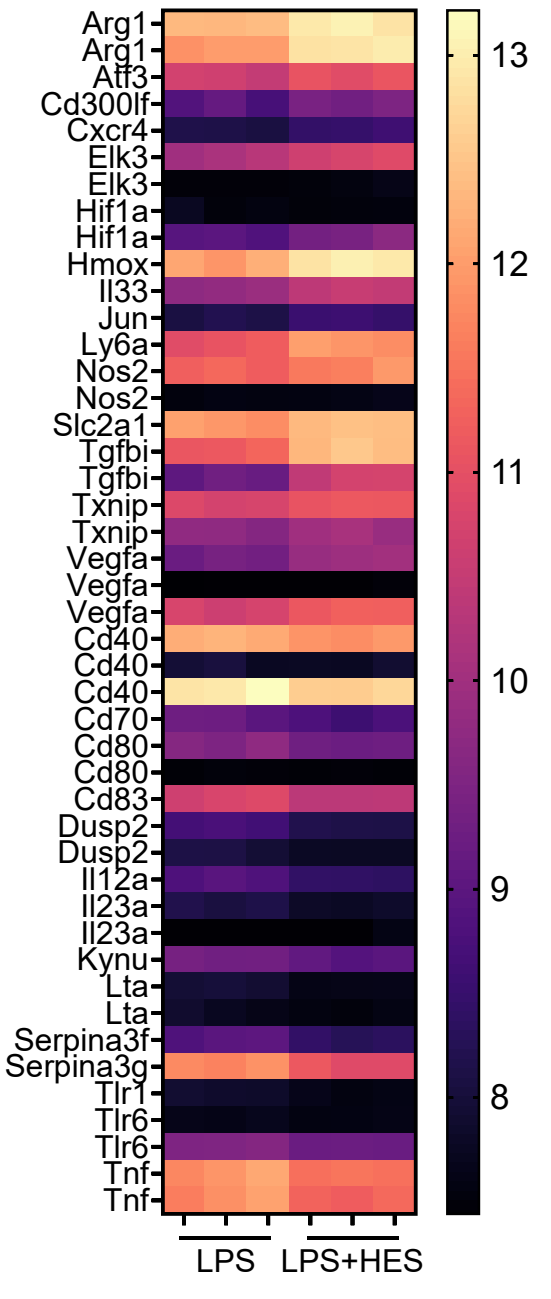

Arg1
Arg1
Atf3
Cd300lf
Cxcr4
Elk3
Elk3
Hif1a
Hif1a
Hmox
Il33
Jun
Ly6a
Nos2
Nos2
Slc2a1
Tgfbi
Tgfbi
Txnip
Txnip
Vegfa
Vegfa
Vegfa
Cd40
Cd40
Cd40
Cd70
Cd80
Cd80
Cd83
Dusp2
Dusp2
Il12a
Il23a
Il23a
Kynu
Lta
Lta
Serpina3f
Serpina3g
Tlr1
Tlr6
Tlr6
Tnf
Tnf
LPS
LPS+HES
13
12
11
10
9
8

Suppl. Fig 3: HES induces a subtle but distinctive change in GMDC gene expression.

Gene expression changes in C57BL/6 GMDC after 8 h of LPS-stimulation with or without HES were measured by microarray. Shown is a heatmap of normalized expression levels (log base 2) for GMDC shown in **Fig. 3A** per individual sample tested, for the transcripts highlighted in that figure. Data are from 3 samples per group from one experiment.
