# Supplementary material for: Curtailment of Toll-like receptor signalling and cytokine production in dendritic cells by secreted products of Heligmosomoides polygyrus
Source: PLoS Pathog. 2026 Aug 26;22(8):e1014528. doi: 10.1371/journal.ppat.1014528 (PMC13577464; doi:10.1371/journal.ppat.1014528)
Supplement: Supplementary file 4 [file ppat.1014528.s004.pdf]

Figure S4

A *C57BL/6 GMDC*

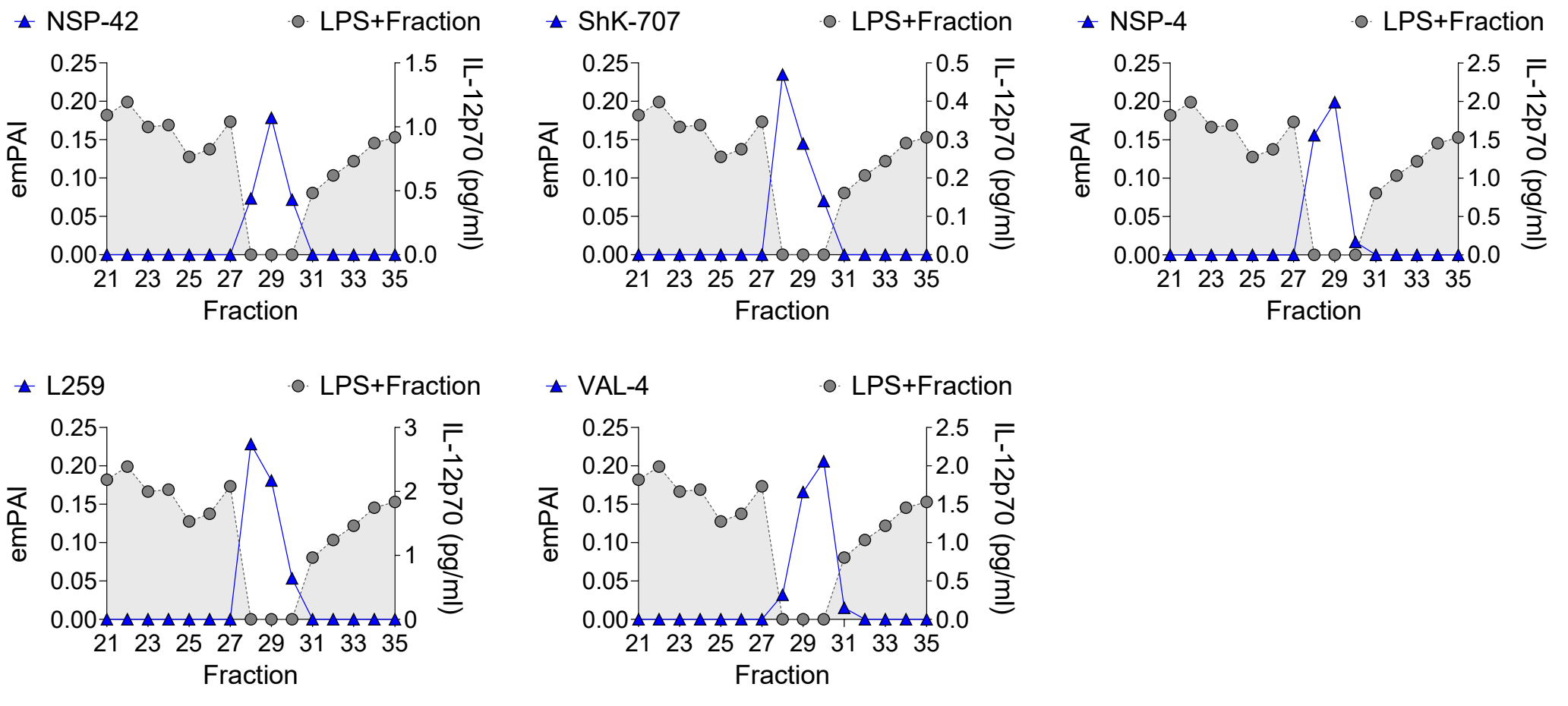

B *C57BL/6 GMDC*

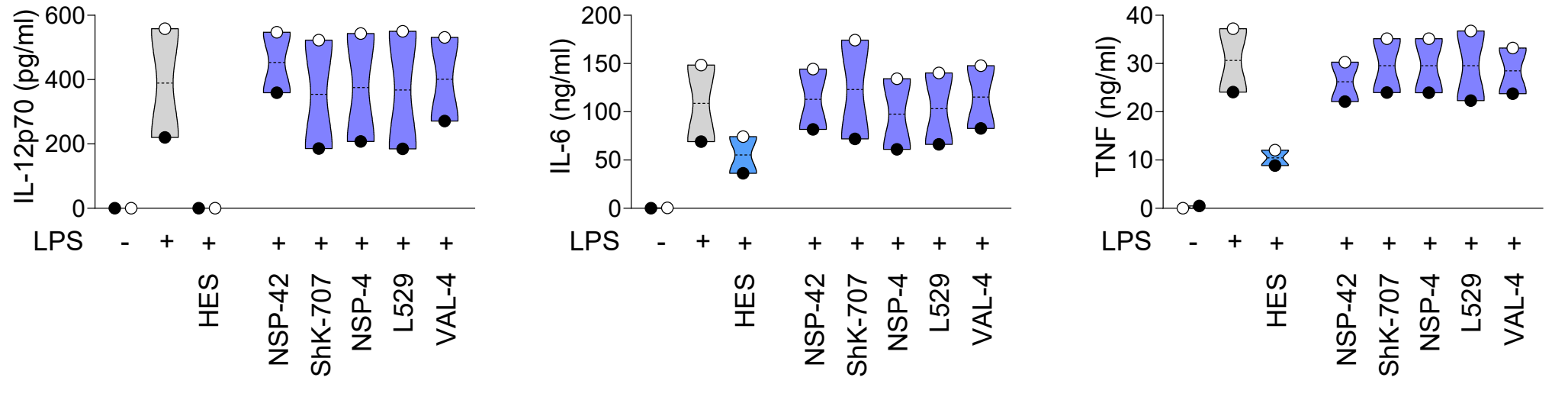

Suppl. Fig 4: The DC-modulator in HES is heat-labile and can be traced to specific chromatography fractions.

A. Chromatographic profile of presence of 5 candidate proteins superimposed on the inhibitory effects of each fraction on IL-12 responses; details of each protein are given in **Suppl. Table 1**.

B. Testing of each candidate as recombinant proteins expressed in mammalian HEK293 cells, in assays for cytokine responses to TLR ligation.
