# Supplementary material for: Curtailment of Toll-like receptor signalling and cytokine production in dendritic cells by secreted products of Heligmosomoides polygyrus
Source: PLoS Pathog. 2026 Aug 26;22(8):e1014528. doi: 10.1371/journal.ppat.1014528 (PMC13577464; doi:10.1371/journal.ppat.1014528)
Supplement: Supplementary file 5 [file ppat.1014528.s005.pdf]

Figure S5

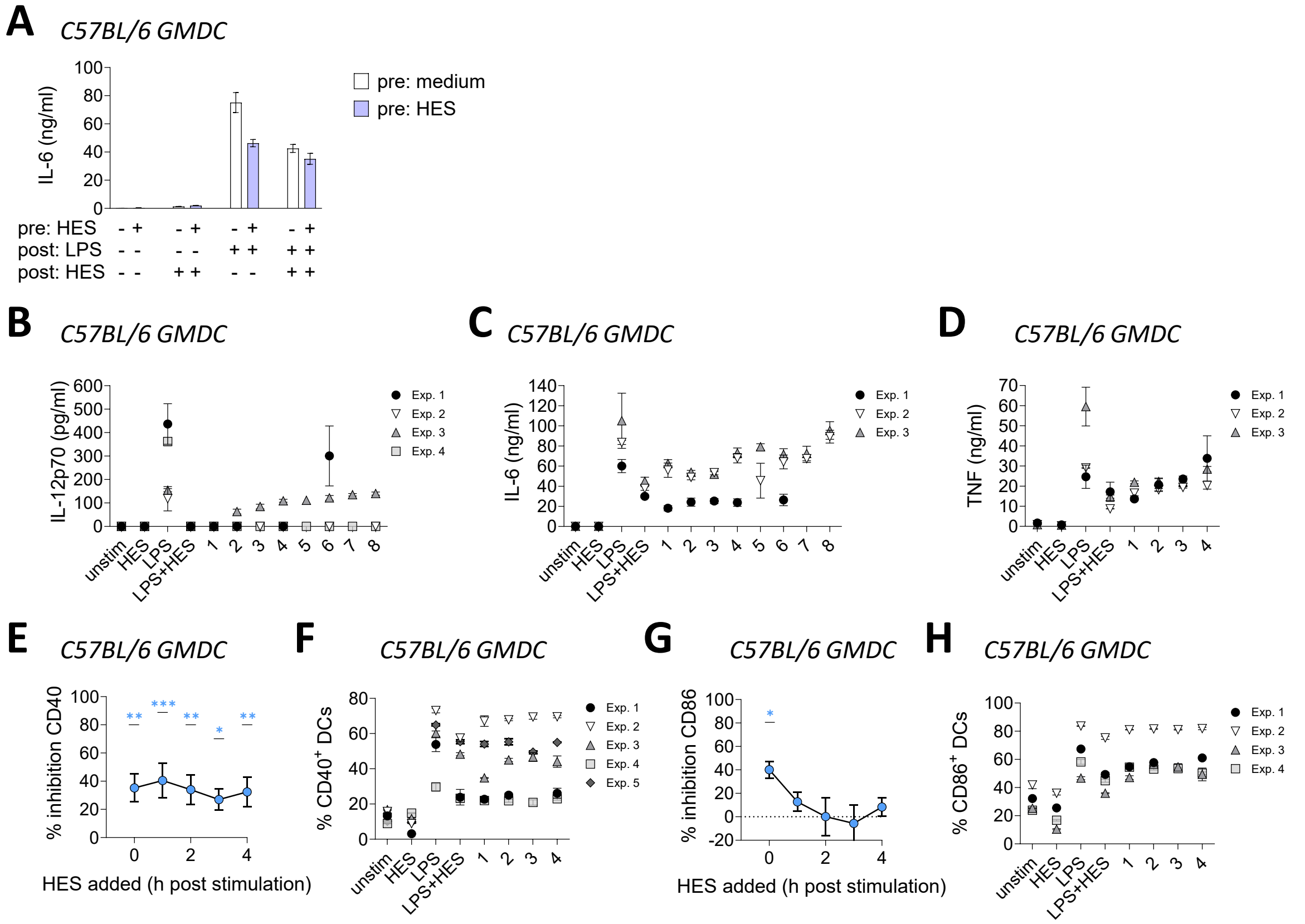
A
C57BL/6 GMDC
IL-6 (ng/ml)
pre: medium
pre: HES
pre: HES - + - + - + - +
post: LPS - - - - + + + +
post: HES - - + + - - + +
B
C57BL/6 GMDC
IL-12p70 (pg/ml)
Exp. 1
Exp. 2
Exp. 3
Exp. 4
unstim HES LPS LPS+HES 1 2 3 4 5 6 7 8
C
C57BL/6 GMDC
IL-6 (ng/ml)
Exp. 1
Exp. 2
Exp. 3
unstim HES LPS LPS+HES 1 2 3 4 5 6 7 8
D
C57BL/6 GMDC
TNF (ng/ml)
Exp. 1
Exp. 2
Exp. 3
unstim HES LPS LPS+HES 1 2 3 4
E
C57BL/6 GMDC
% inhibition CD40
** *** ** * **
HES added (h post stimulation)
F
C57BL/6 GMDC
% CD40+ DCs
Exp. 1
Exp. 2
Exp. 3
Exp. 4
Exp. 5
unstim HES LPS LPS+HES 1 2 3 4
G
C57BL/6 GMDC
% inhibition CD86
*
HES added (h post stimulation)
H
C57BL/6 GMDC
% CD86+ DCs
Exp. 1
Exp. 2
Exp. 3
Exp. 4
unstim HES LPS LPS+HES 1 2 3 4

Suppl. Figure 5: Pre-incubation or post-incubation of GMDC with HES impairs LPS-induced activation.

GMDC of C57BL/6 mice were differentiated with GM-CSF for ten days before treatment as described below.

A. Cells were treated as shown in **Fig. 5 A** and secretion of IL-6 was measured by ELISA. Samples pre-treated with HES are purple bars, stimulation after the washing step is indicated below the x-axis. Each symbol represents the means ± SEM of 3 samples from one experiment.

B-D. Induced levels of cytokines in experimental groups shown as percent inhibition in Fig. 5 D-F following addition of HES at 1-8 hours subsequent to LPS.

E-H Cells were stimulated with LPS, and 10 µg/mL of HES added at the indicated time points after stimulation. Expression of CD40 (**E,F**) and CD86 (**G,H**) on $CD11c^+$ cells after 18 h culture was measured by flow cytometry, converted to percent inhibition (**E,G**) with actual percentage also shown (**F, H**). Data show means ± SEM of 3 averaged samples from 5 (E,F) and 4 (G,H) independent experiments.

In E andG, results from mixed-effects model with Dunnett's multiple comparison test are indicated as * : $p \leq 0.05$; ** : $p \leq 0.01$; *** : $p \leq 0.001$; **** : $p \leq 0.0001$ for the comparison of the LPS stimulated groups (0% inhibition) to each time point.
