# Supplementary material for: Curtailment of Toll-like receptor signalling and cytokine production in dendritic cells by secreted products of Heligmosomoides polygyrus
Source: PLoS Pathog. 2026 Aug 26;22(8):e1014528. doi: 10.1371/journal.ppat.1014528 (PMC13577464; doi:10.1371/journal.ppat.1014528)
Supplement: Supplementary file 6 [file ppat.1014528.s006.pdf]

# Figure S6

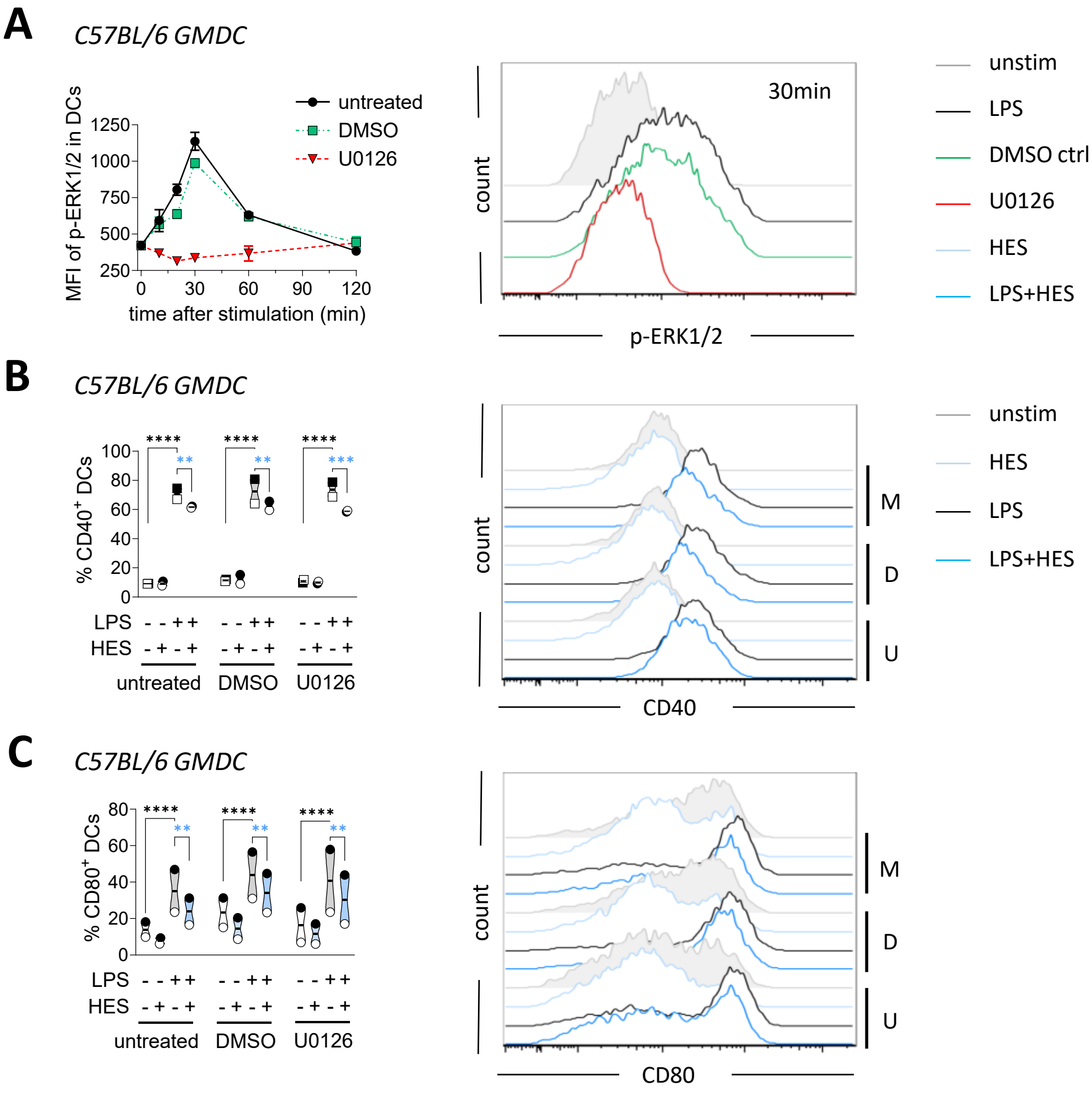
A
C57BL/6 GMDC
MFI of p-ERK1/2 in DCs
1250
1000
750
500
250
0
30
60
90
120
time after stimulation (min)
untreated
DMSO
U0126
count
30min
p-ERK1/2
unstim
LPS
DMSO ctrl
U0126
HES
LPS+HES
B
C57BL/6 GMDC
% CD40+ DCs
100
80
60
40
20
0
****
**
***
LPS - - + +
HES - + - +
untreated
DMSO
U0126
count
CD40
M
D
U
unstim
HES
LPS
LPS+HES
C
C57BL/6 GMDC
% CD80+ DCs
80
60
40
20
0
****
**
LPS - - + +
HES - + - +
untreated
DMSO
U0126
count
CD80
M
D
U

**Suppl. Figure 6: The inhibitory effects of HES are independent of early signalling through ERK, p38, JNK or PI3K**

GMDC were differentiated with GM-CSF for ten days before incubation with cRPMI alone, DMSO in cRPMI or 10 µM U0126 in cRPMI for 60-90 minutes. Following this, cells were stimulated with LPS and HES as indicated.

**A.** Phosphorylation status of ERK1/2 in LPS stimulated cells at the indicated time points was determined by intracellular flow cytometry. Right: p-ERK1/2 in $CD11c^+$ cells at 30min post stimulation.

**B,C.** After 18h of stimulation, expression levels of **(B)** CD40 and **(C)** CD80 on $CD11c^+$ cells were determined by flow cytometry.

Data in **A** show means ± SEM of 3 samples for one experiment. For data in **B, C** each symbol represents the mean of 3 samples from one experiment, symbol colour marking each independent experiment. n = 2 experiments. Results from 2way RM ANOVA and Tukey's multiple comparison test are indicated as * : $p \leq 0.05$; ** : $p \leq 0.01$; *** : $p \leq 0.001$; **** : $p \leq 0.0001$ Sources of variation for the 2-way ANOVA are compiled in Supplementary Information (Statistical and Data Annex).
