# Supplementary material for: Curtailment of Toll-like receptor signalling and cytokine production in dendritic cells by secreted products of Heligmosomoides polygyrus
Source: PLoS Pathog. 2026 Aug 26;22(8):e1014528. doi: 10.1371/journal.ppat.1014528 (PMC13577464; doi:10.1371/journal.ppat.1014528)
Supplement: Supplementary file 7 [file ppat.1014528.s007.pdf]

Figure S7

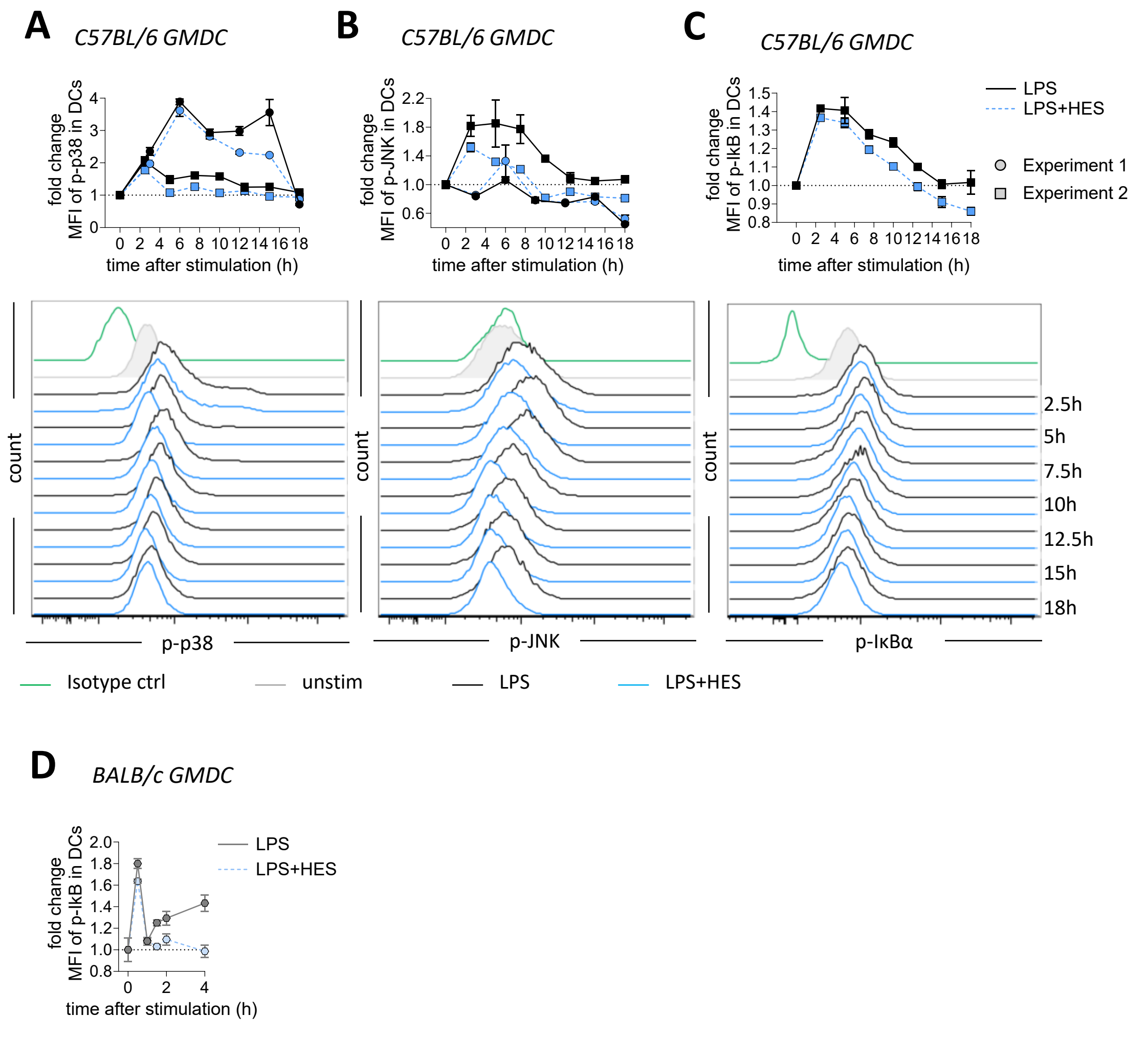
A
C57BL/6 GMDC
fold change MFI of p-p38 in DCs
time after stimulation (h)
B
C57BL/6 GMDC
fold change MFI of p-JNK in DCs
time after stimulation (h)
C
C57BL/6 GMDC
fold change MFI of p-IkB in DCs
time after stimulation (h)
LPS
LPS+HES
Experiment 1
Experiment 2
count
p-p38
p-JNK
p-IκBα
2.5h
5h
7.5h
10h
12.5h
15h
18h
Isotype ctrl
unstim
LPS
LPS+HES
D
BALB/c GMDC
fold change MFI of p-IkB in DCs
time after stimulation (h)
LPS
LPS+HES

Suppl. Figure 7: HES treatment curtails the later stages of signalling.

C57BL/6 GMDC were differentiated with GM-CSF for ten days before stimulation.

A,B. Phosphorylation status of p38 (**A**) and JNK (**B**) in LPS and LPS+HES treated CD11c^{+} cells analysed by flow cytometry. The MFI measured was normalized to unstimulated controls. Data from 2 independent experiments are shown, distinguished by the indicated symbols.

C, D Phosphorylation status of IκBα in LPS and LPS+HES treated GMDC from **(C)** C57BL/6 and **(D)** BALB/c mice was analysed by flow cytometry. The MFI measured was normalized to unstimulated controls. Data show means ± SEM of 3 samples for each experiment.

Results from 2-way ANOVA and Tukey's multiple comparison test are indicated as * : $p \leq 0.05$; ** : $p \leq 0.01$; *** : $p \leq 0.001$; **** : $p \leq 0.0001$; black symbols indicate significance of comparison between LPS-treated cells to the unstimulated controls, blue symbols indicate significance of the comparison between LPS and LPS+HES-treated cells at each time point.

Suppl. Figure 7: HES treatment curtails the later stages of signalling.

C57BL/6 GMDC were differentiated with GM-CSF for ten days before stimulation.

A,B. Phosphorylation status of p38 (**A**) and JNK (**B**) in LPS and LPS+HES treated CD11c^{+} cells analysed by flow cytometry. The MFI measured was normalized to unstimulated controls. Data from 2 independent experiments are shown, distinguished by the indicated symbols.

C, D Phosphorylation status of IκBα in LPS and LPS+HES treated GMDC from **(C)** C57BL/6 and **(D)** BALB/c mice was analysed by flow cytometry. The MFI measured was normalized to unstimulated controls. Data show means ± SEM of 3 samples for each experiment.

Results from 2-way ANOVA and Tukey's multiple comparison test are indicated as * : $p \leq 0.05$; ** : $p \leq 0.01$; *** : $p \leq 0.001$; **** : $p \leq 0.0001$; black symbols indicate significance of comparison between LPS-treated cells to the unstimulated controls, blue symbols indicate significance of the comparison between LPS and LPS+HES-treated cells at each time point.
