# Supplementary material for: Curtailment of Toll-like receptor signalling and cytokine production in dendritic cells by secreted products of Heligmosomoides polygyrus
Source: PLoS Pathog. 2026 Aug 26;22(8):e1014528. doi: 10.1371/journal.ppat.1014528 (PMC13577464; doi:10.1371/journal.ppat.1014528)
Supplement: Supplementary file 8 [file ppat.1014528.s008.pdf]

# Figure S8

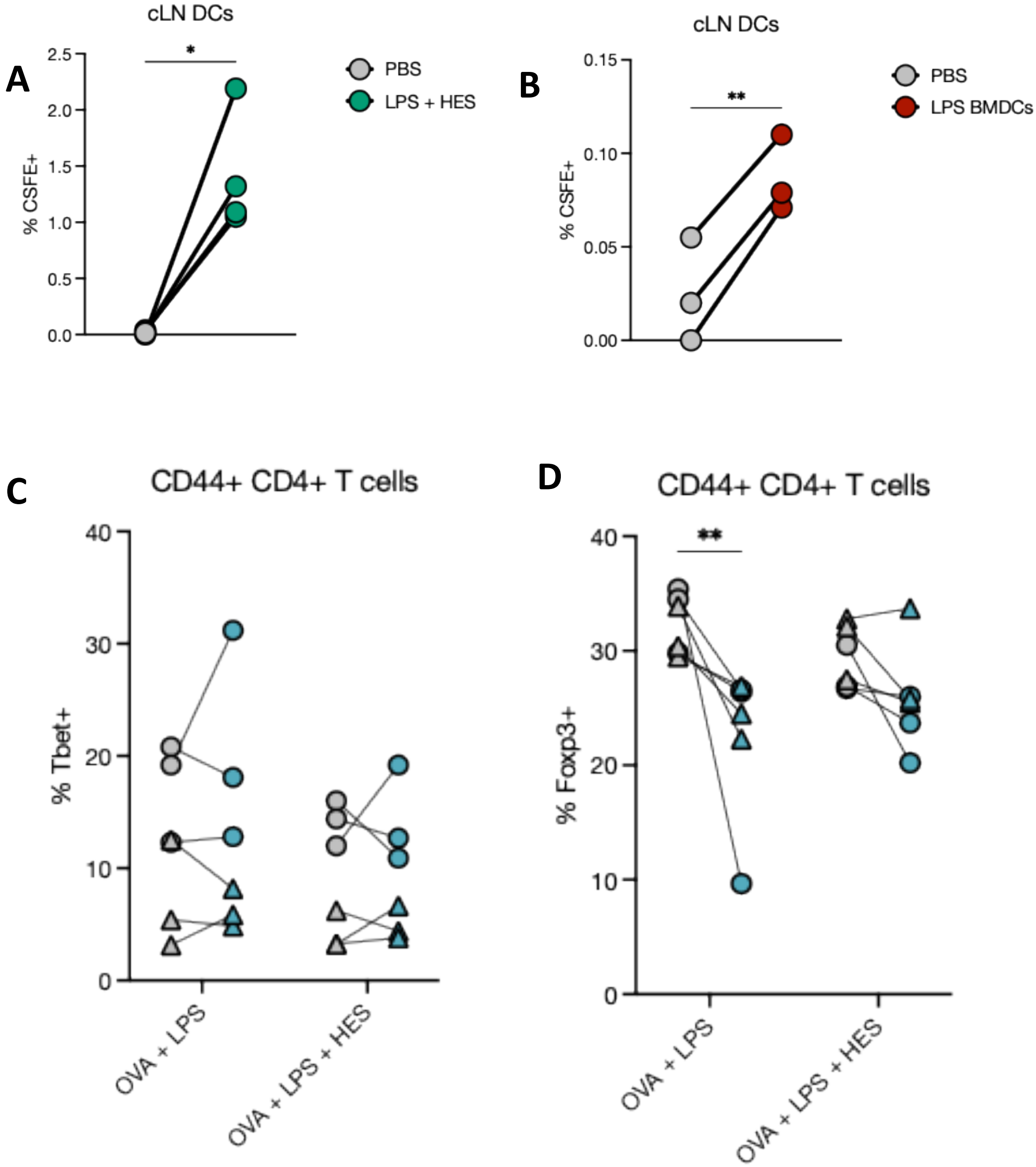
A
cLN DCs
% CSFE+
2.5
2.0
1.5
1.0
0.5
0.0
*
PBS
LPS + HES
B
cLN DCs
% CSFE+
0.15
0.10
0.05
0.00
**
PBS
LPS BMDCs
C
CD44+ CD4+ T cells
% Tbet+
40
30
20
10
0
OVA + LPS
OVA + LPS + HES
D
CD44+ CD4+ T cells
% Foxp3+
40
30
20
10
0
**
OVA + LPS
OVA + LPS + HES

**Suppl. Figure 8: HES-treated DCs mitigate reduction in Foxp3+ Tregs caused byLPS-treated DCs**

GMDC were differentiated with GM-CSF for ten days before stimulation with LPS or LPS+HES for 3 h. $10^6$ GMDC were injected into the ear pinnae of naïve C57BL/6 mice, and draining lymph nodes harvested 24-96h later.

**A-B**. Numbers of labelled GMDC identified in draining lymph nodes of mice injected with LPS+HES-treated (A) or LPS-treated (B) GMDC, compared to the contralateral ear injected with PBS alone.

**C-D.** Percentages of $Tbet^+$ (C) and $Foxp3^+$ (D) $CD44^+$ $CD4^+$ T cells of mice treated as in A and B. Each symbol pair represents one mouse from one experiment performed. n=3-6.
