# Supplementary material for: Curtailment of Toll-like receptor signalling and cytokine production in dendritic cells by secreted products of Heligmosomoides polygyrus
Source: PLoS Pathog. 2026 Aug 26;22(8):e1014528. doi: 10.1371/journal.ppat.1014528 (PMC13577464; doi:10.1371/journal.ppat.1014528)
Supplement: Supplementary file 9 [file ppat.1014528.s009.pdf]

Supplementary Table 1: Candidate proteins tested for inhibition of DC function

Name	# of aa	Mol Wt	Sequence	Accession / Genome numbers	Reference
NSP-42	147	14.17 kDa	MLSTYLTAVCALLTSAGIGANSV RSCSDVTDTVKDTIIRTVEAEVPT LEWNEVFFPYAQQYGINGKKPH PEENYIYLKKKGEFVGNATLDE MATTVLDSVTEETKQALRSNPG NRIYACVAIHCDKTEHTPISLEVA CISTVATMQN	WormBase HPOL_0000224901	(1)
ShK-707	172	17.65 kDa	MLLVFLLLIVVVSGKPLSEGEPQI CEDKSTDCDTQMDVCHDPMWE RAMRRYCAKTCGFCRAEVTTTT TTMTAPPTTSKPHQKPQQQQQQ QSLIILFFQPERCHRLFEQVCRWI VTYGQFPNLIRIPYYNTAQLQRS EASDSDRESNSIPVLGPENDRIFT RPNDSSPTKESD	WormBase HPOL_0000708801	
NSP-4	156	15.78 kDa	MIRFVVSVLSMTHFVSLERINCH GSGGDWQLGMYESYHENLSWY LTLDCDIVGEAIAANEFELKNPN EPQRFLNSTKYCRYSNVLHKDTI GIGGGDVTTLLNGLPREQIKRTL WKLGETTFGCSAHNYRYPAFAD HTHVKLICLYRKQHAEPTCI	WormBase HPOL_0000224901; NCBI CCC54334; EMBL HE573242	(1)
L529	102	10.03 kDa	MLCKLFVLTALICAVASDDEDK GCGPNEKYYEEECAPMETCWSM RVLTRCDKGRCGCKDGYLWGY DGCQLFGTPGCTKEDRLHYSYH THHRKRREAFRFGNH	Not deposited	
VAL-4	213	21.83 kDa	MSTLPTVSFLVVLVALGKAEFGC DGTLEQNDTTREVFLRFHNDVR KFIALGIYPNKVGVLGPAKNMY QLKWSCDLEEEAHESIYSCSYNP LLLHPQSYSKLLSVDLPDTDVVG ATLEMWTEFMRIYGVNTKTNSY NPSFSQFANMAYSKNTKVGCSY KKCGGDTLVTCVYELGVKLPSH PQMWENGPTCVCVAYTDSICND NNLCEYAPTSAR	WormBase HPBE_0002383401; NCBI AEP82919	(1, 2)

Amino acid numbers refer to full-length including predicted signal peptides; Mol.wts are for mature proteins after signal peptide cleavage. Signal peptides predicted by SignalP6.0 are in dark red. Predicted N-linked glycosylation sites (NxS/T except where x is P) are shown in blue. Potential O-lined glycosylation sites (S/T-rich tracts) shown in green. Accession numbers are from NCBI (ncbi.nlm.nih.gov), and genome numbers from WormBase Parasite (parasite.wormbase.org).

1. Hewitson, J. P., Ivens, A. C., Harcus, Y., Filbey, K. J., McSorley, H. J., Murray, J., Bridgett, S., Ashford, D., Dowle, A. A., and Maizels, R. M. (2013) Secretion of protective antigens by tissue-stage nematode larvae revealed by proteomic analysis and vaccination-induced sterile immunity. *PLOS Pathogens* **9**, e1003492
2. Asojo, O. A., Darwiche, R., Gebremedhin, S., Smant, G., Lozano-Torres, J. L., Drurey, C., Pollet, J., Maizels, R. M., Schneiter, R., and Wilbers, R. H. P. (2018) *Heligmosomoides polygyrus* Venom Allergen-like Protein-4 (HpVAL-4) is a sterol binding protein. *Int J Parasitol* **48**, 359-369
