# Supplementary material for: Curtailment of Toll-like receptor signalling and cytokine production in dendritic cells by secreted products of Heligmosomoides polygyrus
Source: PLoS Pathog. 2026 Aug 26;22(8):e1014528. doi: 10.1371/journal.ppat.1014528 (PMC13577464; doi:10.1371/journal.ppat.1014528)
Supplement: Supplementary file 10 [file ppat.1014528.s012.docx]

2 July 2026

The Editors, PLOS Pathogens

**Re :** PPATHOGENS-D-25-03320

**Kemter et al : Curtailment of Toll-like receptor signalling and cytokine production in dendritic cells by secreted products of *Heligmosomoides polygyrus***

Dear Editors,

We thank you and the Reviewers for positive comments and constructive suggestions on our original submission. We are pleased to have revised a much improved manuscript in accordance with the Reviews and present below a point-by-point response.

**Reviewer #1:**

Kemter et al. provide a well written manuscript in which they aim to understand how H. polygyrus derived supernatant (HES) impacts DC (subset) responses to TLR stimuli in vitro / ex vivo. To do so they use stimuli for different TLRs in combination with HES incubation of cells at different timepoints. However, that HES and proteins from other worms are capable to inhibit TLR signals is known. As a readout they analyze targets downstream of TLR signaling of which several were described before to be modulated by HES as also outlined by the authors (IL-12/IL-12p40, CD40, CD80, CD86). They try to define which components of HES modulate the response using a set of methods used previously by an overlapping set of authors (Osbourne M, et al. Immunity. 2017). They were able to assign the inhibitory effect of HES on TLR induced DC activation to a chromatography fraction and find that it is heat sensitive. However, they were unable to relate their observations to specific H. Polygyrus proteins (They just claim that inhibition is TGF-beta independent and therefore not dependent on a known TGF-beta mimic they described - Johnston et al. Nat Commun. 2017). They then focus on timing aspects of HES inhibition and show that HES has inhibitory effects on TLR induced DC activation when added before or some time after LPS stimulation of DCs, which is an interesting and novel aspect. IL-12p70 inhibition notably still worked (somewhat weaker) when HES was added 8hrs post LPS stimulation in contrast to IL-6 and TNF inhibition for which inhibition worked best at earlier timepoints. Towards the end of the manuscript, authors want to make the point that “HES cuts short the lifetime of” (TLR induced) “signaling” and that induction of DC activation would not be impaired by HES in contrast to activation maintenance of DCs. Unfortunately, the data provided in this part is quite variable and seems not strong enough to back authors conclusions.

While authors provide a huge set of experiments, the novelty is mostly limited to timing aspects of DC inhibition. Authors only work with DC cultures and functional / in vivo relevance of their findings is not addressed. Nevertheless, if authors are able to address the substantial points raised below, including provision of additional data supporting their conclusions, their findings would be interesting. Especially that HES is able to inhibit DC activation by pre- or post-treatment could be relevant to understand worm infections or for potential use of HES components (or their mechanisms of action) for drug development.

We thank the Reviewer for this detailed appraisal and would only beg to differ on the question of our studies being “limited to timing aspects of DC inhibition”; in fact, we test a number of signaling pathways, and candidate mediators, that narrow down the likely mechanism(s) of inhibition, albeit as the Reviewer correctly notes, without relating to any specific parasite molecule. We would also highlight that no previous studies have paid attention to the kinetics of inhibition, generally adopting protocols that assume that any effects are immediate or constant over time: our study shows this is not necessarily the case. We also acknowledge that we did not show any in vivo data in our original submission, but address this in point 2.3 below.

**Reviewer #2:**

This study investigates how excretory-secretory (HES) products from the gastrointestinal nematode Heligmosomoides polygyrus modulate dendritic cell (DC) function. The authors demonstrate that HES potently suppresses the secretion of pro-inflammatory cytokines (IL-12p40, IL-6, TNF-α) and the upregulation of co-stimulatory molecules (CD40, CD86, MHCII) in bone marrow-derived DCs (BMDCs) stimulated with various Toll-like receptor (TLR) ligands. Mechanistically, the study reveals that HES does not block the initial phosphorylation of MAPK or NF-κB pathways but instead appears to act further downstream or via a parallel inhibitory pathway. The authors also identify that the suppression is heat-labile and sensitive to proteinase K, suggesting the active factor is a protein. Furthermore, they show that this suppression is independent of the TGF-β mimic (TGM) found in HES, as TGM-depleted HES retains suppressive activity.

The manuscript presents a clear, well-controlled, and significant advance in our understanding of helminth immunomodulation. It effectively rules out several potential mechanisms (e.g., TGF-β mimicry, receptor degradation) and narrows down the active component to a heat-labile protein. The findings are novel and fit well within the scope of PLOS Pathogens. A few clarifications and minor experimental additions would strengthen the paper.

We thank the Reviewer for these positive comments, and also for their suggestions which we address below.

**Reviewer #1: Major comments (our numbering):**

1.1.	**Statistics**: Authors mostly provide data of three biological replicates which is at the lower limit to perform statistical tests. Sometimes they even perform statistics on two replicates like in Fig. 3B, Fig. 4A or 5B-C. Nevertheless, they indicate significance even in cases where it seems hard to appreciate reasonable differences (Like in Fig. 2C). I’m not a statistics expert but I’m not sure if it is appropriate to use the statistical test chosen by the authors. It seems they include data of technical replicates (only shown as mean in figures) and leave out the interaction term of the 2-way ANOVA. To mathematically judge this is beyond my competence. However, even marked as significant, some differences have small effect sizes raising questions about biological relevance of some findings marked as significant (Like in Fig. 2C). Therefore, authors should appreciate effect size (where not already done) in addition to significance throughout the results part (and might state that they used a sensitive test in the statistics part).

First we would like to express our strong agreement that effect size is, biologically, much more important than p value (in particular of course because dynamic parameters that are in the course of changing will be more variable and have lower p values).

With respect to the statistical tests, we appreciate the Reviewer’s concerns and have comprehensively re-analysed all the data presented using more conservative methods. We have reformatted the figures with the revised statistical analyses, as a small number of comparisons now fall outside the statistical threshold. Where appropriate. calculations are now performed on the mean results of the pseudoreplicates of each independent experiment or biological replicate. Where 2-way ANOVA is applied (using the repeated measure ANOVA), we have compiled tables showing the sources of variation including interaction terms, which are now included in a Statistical Annex in the Supplemental Information, to which we refer in the Methods section (lines 622-627) and in the respective Figure Legends.

1.2.	**The Title** “Curtailment of Toll-like receptor signalling and cytokine production in dendritic cells by secreted products of Heligmosomoides polygyrus” does not reflect authors results but is more a description what was done. It should rather reflect authors new findings.

We have mixed feelings about this – the original title is the most straightforward and emphasises that effect is kinetic; we could add details (eg IL-12, or naming the ligands) or include pathways tested, but would result in a more cumbersome title. We hope the Reviewer and Editor will be content with the existing title.

1.3.	Fig. 3: Info about statistical tests in figure legend is missing.

We thank the Reviewer for drawing our attention to this omission; we have now added the information to the Legend for Figure 3 B.

1.4.	Lines 241-242 / Fig. 5D: How can IL-12p70 concentrations be below detection level after HES addition (text) but information like 60% inhibition is shown in the figure?

We thank the Reviewer for pointing out this misworded statement, which we have now amended (Lines 263-264).

1.5.	Fig. 5D-F: To appreciate inhibition it would be good to show induced level of measured products (i.e. in supplement).

We agree with the Reviewer and now present the levels of IL-12p70, IL-6 and TNF measured in each individual experiment in Supplementary Figure 5 B-D.

1.6.	Line 352-353: Why do authors think that “Direct interference with TLR receptor-ligand binding is unlikely as pre-exposure of cells to HES”… “maintained the inhibited phenotype”. Isn’t it possible that HES components bind TLRs and modulate ligand binding that way?

The Reviewer is technically correct, as our experiment only separated HES and the ligands by giving them at separate time points. We have modified our statement (line 383) to indicate that only direct interference with the ligands can be excluded; it is still possible that HES interferes with the TLRs themselves, albeit to 5 separate receptors with similar efficacy.

1.7.	Fig. 7A: The data shows only small effect sizes and only one of two performed experiments supports the authors conclusion of rather late p38 inhibition. Authors should provide additional data to support their conclusion.

The Reviewer is correct in viewing the data in Figure 7 as somewhat marginal in significance. In response we now give less emphasis to this set of experiments and present them in an expanded Suppl. Figure 7, accompanied by a briefer text in the Results section (lines 311-315).

1.8.	Fig. 7B: The data seems quite variable. Further, I have problems to see the “much steeper loss of JNK phosphorylation” (Line 293) the authors propose. The 6 h timepoint which shows the highest. p-JNK values is not even significant versus unstimulated controls. There is also no significance indicated between LPS and LPS+HES groups at any timepoint making it hard to appreciate the authors conclusions. Authors should provide additional data to support their conclusion.

Please see response to 1.7, where this phrase has been modified.

1.9.	Fig. 8: If there are no significant differences between the zero time point and early timepoints in general how should there be significant differences between groups? Which group sizes would be needed to have enough statistical power to reasonably exclude a difference at early timepoints? Based on this, authors should provide sufficient data to support their statements.

The Reviewer refers to the early time points represented by shaded boxes, within which the responses to stimulation have yet to hit a significance cut-off. With the updated statistical analyses, we have removed these boxes (in what is now Figure 7) and amended the main text (lines 333-336 and 349-351)to put less emphasis on significance and instead more on effect sizes, and avoid potentially misleading statements our data is not powered to support.

1.10.	Figure legend titles 7/8 and connected passages in the text: Based on the current data it seems too strong to state that signaling “cuts short” (only backed by one experiment in 7A as 7B seems variable). In addition, “shuts down its maintenance” seems also too strong for figure 8. Based on current data a phrase like “prevents further increase” might be more appropriate.

Following these reasonable comments from the Reviewer we have replaced these phrases with “reduces” and curtails” which are the more appropriate terms (lines 309, 323, 1048).

1.11.	Please add statistics for suppl. Fig. 7A and suppl. Fig. 7B. Otherwise authors should tone down their statements in the results (lines 297-300).

As part of our response to 1.17 and 1.18 we have shortened the text describing Suppl Fig 7 C (was 7A) and D (was 7B) to avoid any over-interpretation (lines 311-320).

**Reviewer #2:** Mechanism of Suppression

The authors show that HES suppresses cytokine production without inhibiting the phosphorylation of p38, ERK, JNK, or NF-κB p65. This is a crucial negative result.
While the authors rule out phosphorylation, they do not address nuclear translocation or DNA binding of these transcription factors. It is possible that HES interferes with the nuclear import or transcriptional activity of NF-κB/AP-1 despite normal phosphorylation.

A brief discussion or a simple immunofluorescence assay for NF-κB p65 nuclear translocation would add mechanistic depth. If not feasible, the discussion should explicitly mention this as a remaining possibility.

The Reviewer raises a very interesting possibility, and one that becomes more likely when considering the ability of some parasite secreted products to enter host cells. We have followed the suggestion of exploring this in the Discussion (lines 425-428).

2.2.	Specificity of the "Suppressor". The study rules out the TGF-β mimic (TGM) as the sole suppressor using specific antibodies. While TGM is ruled out, HES is a complex mixture. Have the authors considered other known immunomodulators like Hp-ARI (Alarmin Release Inhibitor)? Although Hp-ARI targets IL-33, checking if it has any cross-over effect on TLR signaling (or discussing why it wouldn't) would be beneficial.. A brief mention in the discussion regarding other known HES components and why they are unlikely to be the primary drivers of this specific phenotype would be helpful.

We appreciate this suggestion and have now added the point that other known modulators from *H. polygyrus* (ARI, BARI and BoRB) are not present in the active fractions (lines 243-244).

2.3.	In Vivo Relevance. The study relies heavily on in vitro BMDC cultures.
While the in vitro data is robust, correlating this with in vivo DC phenotypes during infection would significantly strengthen the biological relevance.
Do DCs from H. polygyrus-infected mice show a similar refractoriness to TLR stimulation ex vivo? If this data exists or can be easily obtained, it would bridge the gap between the in vitro mechanism and the in vivo infection model.

We thank the Reviewer for raising this important point. We and other laboratories have indeed characterized DCs from infected mice. In Smith et al (2011 J Immunol 186:7098) we found reduced CD40, CD80 on MLN DCs from infected mice and most importantly that CD11c(hi) MLN DCs from infected mice had reduced LPS responses (IL-10 and IL-12) compared to DCs from uninfected mice; we now refer to this in the Introduction (lines 122-124). As this and the other studies originally cited were *ex vivo*, we have now added two further citations in the Introduction to experiments in which DCs from *H. polygyrus*-infected mice were shown by adoptive transfer to confer a tolerogenic outcome (Blum et al. 2012 and Chen et al. 2006) (lines 126-131).

As number of DCs that can be recovered from infected mice is restricted, and as our focus now is on the released parasite products rather than the whole infection, we attempted a new approach of injecting HES-pulsed DCs into the ear pinna and measuring T cell responses in the draining lymph node. While numbers of cells available were modest, we were able to find that HES-treated DCs differed in one important respect, nullifying the loss of Foxp3 Tregs that was observed with LPS-activated DCs. However, the model is still under development and at this stage we would prefer to present the data as a Suppl. Figure (new Suppl. Fig 8), with a brief presentation in the text (lines 352-362) and details in Methods (lines 481-484 and 516-523).

**Reviewer #1 Minor comments:**

1.12	Line 103: Maybe use long version of “pOVA” for clarity.

Yes, we agree better to spell this out which we have now one (lines 118-119).

1.13	Fig. 6: Authors should shortly state limitations of using inhibitors.

We have inserted wording to acknowledge that inhibitor compounds are not always monospecific (lines 288-289).

1.14	Fig. 6: Why authors don’t inhibit p-p38 or p-JNK, while they use inhibitors for MEK and PI3K?

We thank the reviewer for this question; we did not test p38 and JNK inhibitors as these proteins did not show increased phosphorylation at any time (and in fact their patterns in the early phase of activation did not change). We therefore concluded that it was unlikely that inhibition of these signalling pathways would pinpointing the exact target pathways of HES.

1.15	Fig. 6(A, D, E) and Fig. 7: Authors combine raw MFI values from several experiments. However, MFI might vary at different measurement days (variables like staining, flow cytometer…). Data could be normalized to make it more comparable in the line chart.

We agree that normalizing the MFI values is more appropriate when combining data from multiple experiments, and have amended the Figures, statistics and figure legends accordingly.

1.16	Line 413: While authors discuss an interesting aspect here, they should emphasize that measurements in the manuscript (max. ~18 h) would still represent an early timepoint of a worm infection.

The Reviewer raises a pertinent point that we now highlight in the Discussion on lines 449-450.

**Reviewer #2: Minor comments:**

2.4	In Figure 3 (signaling), the western blots are clear, but including densitometry quantification relative to total protein/loading controls would make the "lack of inhibition" claim more quantitative and rigorous.

We are not sure what the Reviewer is referring to here, as we do not show Western blots in any of the Figures.

2.5	The authors state that HES contained low endotoxin levels. Please explicitly state the final concentration of LPS carried over into the assays to ensure it is negligible compared to the TLR4 stimulation doses used.

We now state in the Methods (line 511) the range of endotoxin levels, which are 100-1000 fold lower than those used for LPS stimulation; we would also point to the heat-lability of the HES effect (Figure 4 A,B) and the behaviour of the active principle on ion exchange and size exclusion chromatography, neither of which are compatible with an LPS effect.

2.6	Ensure that the specific post-hoc tests used for multiple comparisons (e.g., in Figure 1 and 2) are clearly stated in every figure legend.

We have checked each legend and where this information was missing (specifically Figure 3 B) it has now been added.

2.7.	This is a solid study that advances the field by characterizing a novel immunosuppressive activity in H. polygyrus secretions that is distinct from the well-known TGF-β mimic. The experiments are logical and the conclusions are supported by the data. Addressing the points above will polish the manuscript for publication.

We very much appreciate the Reviewer’s positive comments and thank them for the constructive suggestions that we have been very pleased to follow.

Yours sincerely,

Prof Rick Maizels FRSE FMedSci
