# Supplementary material for: Curtailment of Toll-like receptor signalling and cytokine production in dendritic cells by secreted products of Heligmosomoides polygyrus
Source: PLoS Pathog. 2026 Aug 26;22(8):e1014528. doi: 10.1371/journal.ppat.1014528 (PMC13577464; doi:10.1371/journal.ppat.1014528)
Supplement: Peer review history

# Peer review: Decision Letter 0

**DOI:** 10.1371/journal.ppat.1014528.r001

**Authors:** Edward Mitre, Mohamed Abouelkhair

24 Feb 2026

Curtailment of Toll-like receptor signalling and cytokine production in dendritic cells by secreted products of Heligmosomoides polygyrus

PLOS Pathogens

Dear Dr. Maizels,

Thank you for submitting your manuscript to PLOS Pathogens. After careful consideration, we feel that it has merit but does not fully meet PLOS Pathogens's publication criteria as it currently stands. Therefore, we invite you to submit a revised version of the manuscript that addresses the points raised during the review process. In its current form, the mechanistic depth and in vivo relevance remain limited. While the in vitro data are extensive, the conclusions regarding the mechanism of suppression and the impact on signaling maintenance are not sufficiently supported. In particular, additional mechanistic clarification would strengthen the study, and further data linking the in vitro observations to dendritic cell function during infection would substantially enhance the biological relevance of the work.

Please submit your revised manuscript by Apr 25 2026 11:59PM. If you will need more time than this to complete your revisions, please reply to this message or contact the journal office at plospathogens@plos.org. When you're ready to submit your revision, log on to https://www.editorialmanager.com/ppathogens/ and select the 'Submissions Needing Revision' folder to locate your manuscript file.

Please include the following items when submitting your revised manuscript:

* A letter that responds to each point raised by the editor and reviewer(s). You should upload this letter as a separate file labeled 'Response to Reviewers'. This file does not need to include responses to any formatting updates and technical items listed in the 'Journal Requirements' section below.

* A marked-up copy of your manuscript that highlights changes made to the original version. You should upload this as a separate file labeled 'Revised Manuscript with Track Changes'.

* An unmarked version of your revised paper without tracked changes. You should upload this as a separate file labeled 'Manuscript'.

If you would like to make changes to your financial disclosure, competing interests statement, or data availability statement, please make these updates within the submission form at the time of resubmission. Guidelines for resubmitting your figure files are available below the reviewer comments at the end of this letter.

We look forward to receiving your revised manuscript.

Kind regards,

Mohamed A Abouelkhair, Ph.D., MS, DVM, DACVM

Academic Editor

PLOS Pathogens

Edward Mitre

Section Editor

Sumita Bhaduri-McIntosh

Editor-in-Chief

PLOS Pathogens

orcid.org/0000-0003-2946-9497

Michael Malim

PLOS Pathogens

orcid.org/0000-0002-7699-2064

**Journal Requirements:**

If the reviewer comments include a recommendation to cite specific previously published works, please review and evaluate these publications to determine whether they are relevant and should be cited. There is no requirement to cite these works unless the editor has indicated otherwise.

1) Please provide an Author Summary. This should appear in your manuscript between the Abstract (if applicable) and the Introduction, and should be 150-200 words long. The aim should be to make your findings accessible to a wide audience that includes both scientists and non-scientists. Sample summaries can be found on our website under Submission Guidelines:

https://journals.plos.org/plospathogens/s/submission-guidelines#loc-parts-of-a-submission

2) We do not publish any copyright or trademark symbols that usually accompany proprietary names, eg ©,  ®, or TM  (e.g. next to drug or reagent names). Therefore please remove all instances of trademark/copyright symbols throughout the text, including:

- ® on pages: 18, and 19.

3) Please upload all main figures as separate Figure files in .tif or .eps format. For more information about how to convert and format your figure files please see our guidelines:

https://journals.plos.org/plospathogens/s/figures

4) We have noticed that you have uploaded Supporting Information files, but you have not included a list of legends. Please add a full list of legends for your Supporting Information files after the references list.

5) In the online submission form, you indicated that your data will be submitted to a repository upon acceptance. We strongly recommend all authors deposit their data before acceptance, as the process can be lengthy and hold up publication timelines. Please note that, though access restrictions are acceptable now, your entire minimal dataset will need to be made freely accessible if your manuscript is accepted for publication. This policy applies to all data except where public deposition would breach compliance with the protocol approved by your research ethics board. If you are unable to adhere to our open data policy, please kindly revise your statement to explain your reasoning and we will seek the editor's input on an exemption.

6) In the online submission form, you indicated that other original data are available on reasonable request to the corresponding author.. All PLOS journals now require all data underlying the findings described in their manuscript to be freely available to other researchers, either

1. In a public repository

2. Within the manuscript itself

3. Uploaded as supplementary information.

This policy applies to all data except where public deposition would breach compliance with the protocol approved by your research ethics board. If your data cannot be made publicly available for ethical or legal reasons (e.g., public availability would compromise patient privacy), please explain your reasons by return email and your exemption request will be escalated to the editor for approval. Your exemption request will be handled independently and will not hold up the peer review process, but will need to be resolved should your manuscript be accepted for publication. One of the Editorial team will then be in touch if there are any issues.

7) Please amend your detailed Financial Disclosure statement. This is published with the article. It must therefore be completed in full sentences and contain the exact wording you wish to be published.

1) State the initials, alongside each funding source, of each author to receive each grant. For example: "This work was supported by the National Institutes of Health (####### to AM; ###### to CJ) and the National Science Foundation (###### to AM)."

2) State what role the funders took in the study. If the funders had no role in your study, please state: "The funders had no role in study design, data collection and analysis, decision to publish, or preparation of the manuscript."

3) If any authors received a salary from any of your funders, please state which authors and which funders..

If you did not receive any funding for this study, please simply state: u201cThe authors received no specific funding for this work.u201d

8)  Please ensure that the funders and grant numbers match between the Financial Disclosure field and the Funding Information tab in your submission form. Note that the funders must be provided in the same order in both places as well.

**Reviewers' Comments:**

Reviewer's Responses to Questions

**Part I - Summary**

Please use this section to discuss strengths/weaknesses of study, novelty/significance, general execution and scholarship.

Reviewer #1: Kempter et al. provide a well written manuscript in which they aim to understand how H. Polygyrus derived supernatant (HES) impacts DC (subset) responses to TLR stimuli in vitro / ex vivo. To do so they use stimuli for different TLRs in combination with HES incubation of cells at different timepoints. However, that HES and proteins from other worms are capable to inhibit TLR signals is known. As a readout they analyze targets downstream of TLR signaling of which several were described before to be modulated by HES as also outlined by the authors (IL-12/IL-12p40, CD40, CD80, CD86). They try to define which components of HES modulate the response using a set of methods used previously by an overlapping set of authors (Osbourn M, et al. Immunity. 2017). They were able to assign the inhibitory effect of HES on TLR induced DC activation to a chromatography fraction and find that it is heat sensitive. However, they were unable to relate their observations to specific H. Polygyrus proteins (They just claim that inhibition is TGF-beta independent and therefore not dependent on a known TGF-beta mimic they described - Johnston et al. Nat Commun. 2017). They then focus on timing aspects of HES inhibition and show that HES has inhibitory effects on TLR induced DC activation when added before or some time after LPS stimulation of DCs, which is an interesting and novel aspect. IL-12p70 inhibition notably still worked (somewhat weaker) when HES was added 8hrs post LPS stimulation in contrast to IL-6 and TNF inhibition for which inhibition worked best at earlier timepoints. Towards the end of the manuscript, authors want to make the point that “HES cuts short the lifetime of” (TLR induced) “signaling” and that induction of DC activation would not be impaired by HES in contrast to activation maintenance of DCs. Unfortunately, the data provided in this part is quite variable and seems not strong enough to back authors conclusions.

While authors provide a huge set of experiments, the novelty is mostly limited to timing aspects of DC inhibition. Authors only work with DC cultures and functional / in vivo relevance of their findings is not addressed. Nevertheless, if authors are able to address the substantial points raised below, including provision of additional data supporting their conclusions, their findings would be interesting. Especially that HES is able to inhibit DC activation by pre- or post-treatment could be relevant to understand worm infections or for potential use of HES components (or their mechanisms of action) for drug development.

Reviewer #2: This study investigates how excretory-secretory (HES) products from the gastrointestinal nematode Heligmosomoides polygyrus modulate dendritic cell (DC) function. The authors demonstrate that HES potently suppresses the secretion of pro-inflammatory cytokines (IL-12p40, IL-6, TNF-α) and the upregulation of co-stimulatory molecules (CD40, CD86, MHCII) in bone marrow-derived DCs (BMDCs) stimulated with various Toll-like receptor (TLR) ligands. Mechanistically, the study reveals that HES does not block the initial phosphorylation of MAPK or NF-κB pathways but instead appears to act further downstream or via a parallel inhibitory pathway. The authors also identify that the suppression is heat-labile and sensitive to proteinase K, suggesting the active factor is a protein. Furthermore, they show that this suppression is independent of the TGF-β mimic (TGM) found in HES, as TGM-depleted HES retains suppressive activity.

The manuscript presents a clear, well-controlled, and significant advance in our understanding of helminth immunomodulation. It effectively rules out several potential mechanisms (e.g., TGF-β mimicry, receptor degradation) and narrows down the active component to a heat-labile protein. The findings are novel and fit well within the scope of PLOS Pathogens. A few clarifications and minor experimental additions would strengthen the paper.

**********

**Part II – Major Issues: Key Experiments Required for Acceptance**

Please use this section to detail the key new experiments or modifications of existing experiments that should be absolutely required to validate study conclusions.

Generally, there should be no more than 3 such required experiments or major modifications for a "Major Revision" recommendation. If more than 3 experiments are necessary to validate the study conclusions, then you are encouraged to recommend "Reject".

Reviewer #1: Major comments:

- Statistics: Authors mostly provide data of three biological replicates which is at the lower limit to perform statistical tests. Sometimes they even perform statistics on two replicates like in Fig. 3B, Fig. 4A or 5B-C. Nevertheless, they indicate significance even in cases where it seems hard to appreciate reasonable differences (Like in Fig. 2C). I’m not a statistics expert but I’m not sure if it is appropriate to use the statistical test chosen by the authors. It seems they include data of technical replicates (only shown as mean in figures) and leave out the interaction term of the 2-way ANOVA. To mathematically judge this is beyond my competence. However, even marked as significant, some differences have small effect sizes raising questions about biological relevance of some findings marked as significant (Like in Fig. 2C). Therefore, authors should appreciate effect size (where not already done) in addition to significance throughout the results part (and might state that they used a sensitive test in the statistics part).

- The title “Curtailment of Toll-like receptor signalling and cytokine production in dendritic cells by secreted products of Heligmosomoides polygyrus” does not reflect authors results but is more a description what was done. It should rather reflect authors new findings.

- Fig. 3: Info about statistical tests in figure legend is missing.

- Lines 241-242 / Fig. 5D: How can IL-12p70 concentrations be below detection level after HES addition (text) but information like 60% inhibition is shown in the figure?

- Fig. 5D-F: To appreciate inhibition it would be good to show induced level of measured products (i.e. in supplement).

- Line 352-353: Why do authors think that “Direct interference with TLR receptor-ligand binding is unlikely as pre-exposure of cells to HES”… “maintained the inhibited phenotype”. Isn’t it possible that HES components bind TLRs and modulate ligand binding that way?

- Fig. 7A: The data shows only small effect sizes and only one of two performed experiments supports the authors conclusion of rather late p38 inhibition. Authors should provide additional data to support their conclusion.

- Fig. 7B: The data seems quite variable. Further, I have problems to see the “much steeper loss of JNK phosphorylation” (Line 293) the authors propose. The 6 h timepoint which shows the highest p-JNK values is not even significant versus unstimulated controls. There is also no significance indicated between LPS and LPS+HES groups at any timepoint making it hard to appreciate the authors conclusions. Authors should provide additional data to support their conclusion.

- Fig. 8: If there are no significant differences between the zero time point and early timepoints in general how should there be significant differences between groups? Which group sizes would be needed to have enough statistical power to reasonably exclude a difference at early timepoints? Based on this, authors should provide sufficient data to support their statements.

- Figure legend titles 7/8 and connected passages in the text: Based on the current data it seems too strong to state that signaling “cuts short” (only backed by one experiment in 7A as 7B seems variable). In addition, “shuts down its maintenance” seems also too strong for figure 8. Based on current data a phrase like “prevents further increase” might be more appropriate.

- Please add statistics for suppl. Fig. 7A and suppl. Fig. 7B. Otherwise authors should tone down their statements in the results (lines 297-300).

Reviewer #2: Mechanism of Suppression

The authors show that HES suppresses cytokine production without inhibiting the phosphorylation of p38, ERK, JNK, or NF-κB p65. This is a crucial negative result.

While the authors rule out phosphorylation, they do not address nuclear translocation or DNA binding of these transcription factors. It is possible that HES interferes with the nuclear import or transcriptional activity of NF-κB/AP-1 despite normal phosphorylation.

A brief discussion or a simple immunofluorescence assay for NF-κB p65 nuclear translocation would add mechanistic depth. If not feasible, the discussion should explicitly mention this as a remaining possibility.

Specificity of the "Suppressor"

The study rules out the TGF-β mimic (TGM) as the sole suppressor using specific antibodies.

While TGM is ruled out, HES is a complex mixture. Have the authors considered other known immunomodulators like Hp-ARI (Alarmin Release Inhibitor)? Although Hp-ARI targets IL-33, checking if it has any cross-over effect on TLR signaling (or discussing why it wouldn't) would be beneficial.

A brief mention in the discussion regarding other known HES components and why they are unlikely to be the primary drivers of this specific phenotype would be helpful.

In Vivo Relevance

The study relies heavily on in vitro BMDC cultures.

While the in vitro data is robust, correlating this with in vivo DC phenotypes during infection would significantly strengthen the biological relevance.

Do DCs from H. polygyrus-infected mice show a similar refractoriness to TLR stimulation ex vivo? If this data exists or can be easily obtained, it would bridge the gap between the in vitro mechanism and the in vivo infection model.

**********

**Part III – Minor Issues: Editorial and Data Presentation Modifications**

Please use this section for editorial suggestions as well as relatively minor modifications of existing data that would enhance clarity.

Reviewer #1: Minor comments:

- Line 103: Maybe use long version of “pOVA” for clarity.

- Fig. 6: Authors should shortly state limitations of using inhibitors.

- Fig. 6: Why authors don’t inhibit p-p38 or p-JNK, while they use inhibitors for MEK and PI3K?

- Fig. 6(A, D, E) and Fig. 7: Authors combine raw MFI values from several experiments. However, MFI might vary at different measurement days (variables like staining, flow cytometer…). Data could be normalized to make it more comparable in the line chart.

- Line 413: While authors discuss an interesting aspect here, they should emphasize that measurements in the manuscript (max. ~18 h) would still represent an early timepoint of a worm infection.

Reviewer #2: In Figure 3 (signaling), the western blots are clear, but including densitometry quantification relative to total protein/loading controls would make the "lack of inhibition" claim more quantitative and rigorous.

The authors state that HES contained low endotoxin levels. Please explicitly state the final concentration of LPS carried over into the assays to ensure it is negligible compared to the TLR4 stimulation doses used.

Ensure that the specific post-hoc tests used for multiple comparisons (e.g., in Figure 1 and 2) are clearly stated in every figure legend.

This is a solid study that advances the field by characterizing a novel immunosuppressive activity in H. polygyrus secretions that is distinct from the well-known TGF-β mimic. The experiments are logical and the conclusions are supported by the data. Addressing the points above will polish the manuscript for publication.

**********

PLOS authors have the option to publish the peer review history of their article (what does this mean?). If published, this will include your full peer review and any attached files.

If you choose “no”, your identity will remain anonymous but your review may still be made public.

**Do you want your identity to be public for this peer review?**  For information about this choice, including consent withdrawal, please see our Privacy Policy.

Reviewer #1: No

Reviewer #2: No

**Figure resubmission:**

**Reproducibility:**

?>

# Author comment: Author response to Decision Letter 1

**DOI:** 10.1371/journal.ppat.1014528.r002

8 Jul 2026

Attachment Submitted filename: Kemter-Response-to-Reviewers-2026-07-02.docx

# Peer review: Decision Letter 1

**DOI:** 10.1371/journal.ppat.1014528.r003

**Authors:** Edward Mitre, Mohamed Abouelkhair

6 Aug 2026

Dear Prof. Maizels,

We are pleased to inform you that your manuscript 'Curtailment of Toll-like receptor signalling and cytokine production in dendritic cells by secreted products of Heligmosomoides polygyrus' has been provisionally accepted for publication in PLOS Pathogens.

Before your manuscript can be formally accepted you will need to complete some formatting changes, which you will receive in a follow up email. A member of our team will be in touch with a set of requests.

Please note that your manuscript will not be scheduled for publication until you have made the required changes, so a swift response is appreciated.

IMPORTANT: The editorial review process is now complete. PLOS will only permit corrections to spelling, formatting or significant scientific errors from this point onwards. Requests for major changes, or any which affect the scientific understanding of your work, will cause delays to the publication date of your manuscript.

Should you, your institution's press office or the journal office choose to press release your paper, you will automatically be opted out of early publication. We ask that you notify us now if you or your institution is planning to press release the article. All press must be co-ordinated with PLOS.

Thank you again for supporting Open Access publishing; we are looking forward to publishing your work in PLOS Pathogens.

Best regards,

Mohamed A Abouelkhair, Ph.D., MS, DVM, DACVM

Academic Editor

PLOS Pathogens

Edward Mitre

Section Editor

PLOS Pathogens

Sumita Bhaduri-McIntosh

Editor-in-Chief

PLOS Pathogens

orcid.org/0000-0003-2946-9497

Michael Malim

Editor-in-Chief

PLOS Pathogens

orcid.org/0000-0002-7699-2064

***********************************************************

Reviewer Comments (if any, and for reference):

Reviewer's Responses to Questions

**Part I - Summary**

Please use this section to discuss strengths/weaknesses of study, novelty/significance, general execution and scholarship.

Reviewer #1: The authors have significantly improved the manuscript, refining their arguments and strengthening the overall quality of the paper. Kemter et al. provide a detailed investigation into how HES modulates DC responses to TLR stimuli. By utilizing chromatography and timing-based assays, they successfully isolate heat-sensitive components that inhibit signaling in a TGF-beta independent manner. The most novel aspect of the work is the characterization of the timing of these effects; specifically, the differing windows of inhibition for IL-12p70 versus IL-6 and TNF. While the study remains mainly focused on in vitro models, it provides a comprehensive look at how HES influences the duration of DC activation. In my opinion, the manuscript is now suitable for publication.

Reviewer #2: The revised manuscript is substantially improved. The authors have made commendable efforts to address the reviewers' concerns, particularly by reigning in overreaching mechanistic claims, providing necessary in vivo context, and completely overhauling their statistical reporting. The paper now presents a highly robust, measured, and compelling story about how HES selectively interrupts the maintenance phase of DC activation. The decision to comprehensively re-analyze all data using more conservative statistical methods is excellent.

**********

**Part II – Major Issues: Key Experiments Required for Acceptance**

Please use this section to detail the key new experiments or modifications of existing experiments that should be absolutely required to validate study conclusions.

Generally, there should be no more than 3 such required experiments or major modifications for a "Major Revision" recommendation. If more than 3 experiments are necessary to validate the study conclusions, then you are encouraged to recommend "Reject".

Reviewer #1: (No Response)

Reviewer #2: None.

**********

**Part III – Minor Issues: Editorial and Data Presentation Modifications**

Please use this section for editorial suggestions as well as relatively minor modifications of existing data that would enhance clarity.

Reviewer #1: (No Response)

Reviewer #2: None.

**********

PLOS authors have the option to publish the peer review history of their article (what does this mean?). If published, this will include your full peer review and any attached files.

If you choose “no”, your identity will remain anonymous but your review may still be made public.

**Do you want your identity to be public for this peer review?**  For information about this choice, including consent withdrawal, please see our Privacy Policy.

Reviewer #1: **Yes:** Daniel Radtke

Reviewer #2: No

# Editor report: Acceptance letter

**DOI:** 10.1371/journal.ppat.1014528.r004

**Authors:** Edward Mitre, Mohamed Abouelkhair

Dear Prof. Maizels,

We are delighted to inform you that your manuscript, "Curtailment of Toll-like receptor signalling and cytokine production in dendritic cells by secreted products of Heligmosomoides polygyrus," has been formally accepted for publication in PLOS Pathogens.

We have now passed your article onto the PLOS Production Department who will complete the rest of the pre-publication process. All authors will receive a confirmation email upon publication.

Thank you again for supporting open-access publishing; we are looking forward to publishing your work in PLOS Pathogens.

Best regards,

Dominique Soldati-Favre

Editor-in-Chief

PLOS Pathogens
